# ABCG transporters are involved in the accumulation of specialized metabolites in the oil bodies of *Marchantia polymorpha*

**DOI:** 10.1093/pcp/pcag069

**Published:** 2026-05-23

**Authors:** Takehiko Kanazawa, Tomoko Kawaguchi, Yuta Moriwaki, Kan-ichi Iwasaki, Takashi Ueda, Kenji Matsui

**Affiliations:** Division of Cellular Dynamics, National Institute for Basic Biology, Nishigonaka 38, Myodaiji, Okazaki, Aichi 444-8585, Japan; Basic Biology Program, Department of Advanced Studies, SOKENDAI (The Graduate University for Advanced Studies), Nishigonaka 38, Myodaiji, Okazaki, Aichi 444-8585, Japan; Department of Biological Chemistry, Faculty of Agriculture and Graduate School of Sciences and Technology for Innovation, Yamaguchi University, Yoshida 1677-1, Yamaguchi, Yamaguchi 753-8515, Japan; Department of Biological Chemistry, Faculty of Agriculture and Graduate School of Sciences and Technology for Innovation, Yamaguchi University, Yoshida 1677-1, Yamaguchi, Yamaguchi 753-8515, Japan; Department of Biological Chemistry, Faculty of Agriculture and Graduate School of Sciences and Technology for Innovation, Yamaguchi University, Yoshida 1677-1, Yamaguchi, Yamaguchi 753-8515, Japan; Division of Cellular Dynamics, National Institute for Basic Biology, Nishigonaka 38, Myodaiji, Okazaki, Aichi 444-8585, Japan; Basic Biology Program, Department of Advanced Studies, SOKENDAI (The Graduate University for Advanced Studies), Nishigonaka 38, Myodaiji, Okazaki, Aichi 444-8585, Japan; Department of Biological Chemistry, Faculty of Agriculture and Graduate School of Sciences and Technology for Innovation, Yamaguchi University, Yoshida 1677-1, Yamaguchi, Yamaguchi 753-8515, Japan

**Keywords:** ABCG transporter, *Marchantia polymorpha*, oil body, marchantin, sesquiterpene, specialized metabolite

## Abstract

Bioactive specialized metabolites (SMs) are synthesized and sequestered in specific cellular compartments or organelles as a self-defense strategy against their intrinsic toxicity. Liverwort-specific oil bodies accumulate large amounts of SMs and contribute to chemical defense; however, the molecular mechanisms underlying SM sequestration in oil bodies remain largely unknown. Therefore, in this study, we focused on MpABCG1 and MpABCG36, which are ATP-binding cassette (ABC) protein family members localized to the oil bodies of liverwort *Marchantia polymorpha*. Sesquiterpene (thujopsene, chamigrene, and himachalene) accumulation was reduced in the Mp*abcg1* and Mp*abcg36* loss-of-function mutants. Notably, levels of the bisbibenzyls, marchantins C and A, were predominantly reduced in Mp*abcg1*, but not in Mp*abcg36*. Although the Mp*abcg1* mutant formed a number of oil bodies labeled with mCitrine–MpSYP12B (an oil body membrane marker) comparable to that of the wild type, the number of oil bodies stained with BODIPY 493/503, which has an affinity for lipophilic SMs, was reduced. This finding suggests that Mp*ABCG1* and Mp*ABCG36* mutations affect SM accumulation in the oil body but have little impact on oil body formation. Overall, our results highlight the involvement of Mp*ABCG1* and Mp*ABCG36* in the accumulation of SMs and/or their precursors in liverwort oil bodies.

## Introduction

Plants produce and accumulate various specialized metabolites (SMs), such as sesquiterpenoids, flavonoids, and alkaloids. Highly bioactive compounds that defend against herbivores and pathogens often exert toxic or harmful effects on plants themselves. Therefore, SM biosynthesis and accumulation are strictly and often spatiotemporally regulated (Tissier 2012, Widhalm et al. 2015, Blomstedt et al. 2016). SMs and/or their precursors synthesized in plastids or the cytosol are transported and stored in specific organelles and compartments, such as the vacuole and extracellular space, *via* membrane transporters and/or membrane trafficking systems (Ichino and Yazaki 2022).

Among bryophytes (hornworts, mosses, and liverworts), >90% of liverwort species possess a unique organelle called an oil body, in which various SMs accumulate (Suire et al. 2000, Asakawa et al. 2013, He et al. 2013, Tanaka et al. 2016, Romani et al. 2022). Importantly, liverwort oil bodies are structurally and functionally distinct from the oil bodies of seed plants (also known as lipid bodies, lipid droplets, or oleosomes). Whereas seed plant oil bodies/lipid droplets primarily store neutral lipids and arise by budding from a subdomain of the endoplasmic reticulum (ER), liverwort oil bodies are membrane-bound organelles formed through redirection of the secretory pathway (Huang 1992, Kanazawa et al. 2020). In *Marchantia polymorpha*, oil bodies are formed in idioblastic “oil body cells,” which accumulate unique sesquiterpenes (e.g. thujopsene, chamigrene, and himachalene) and bisbibenzyls (marchantin A), as revealed *via* single-cell extraction using microcapillaries, followed by direct mass spectrometry analysis (Tanaka et al. 2016). Thus, in contrast to general lipid droplets, liverwort oil bodies are thought to function as specialized compartments for the sequestration of bioactive metabolites. Several transcription factors, including MpTGA, MpC1HDZ, MpMYC, MpERF13, and MpMYB02, act as key regulators of oil body cell differentiation, oil body formation and maturation, and SM-related enzyme expression (Peñuelas et al. 2019, Kanazawa et al. 2020, Romani et al. 2020, Wang et al. 2023, Gutsche et al. 2024, Kubo et al. 2024). Intermediates and/or final products of oil body SMs are possibly biosynthesized in the cytosol. However, the molecular mechanisms underlying SM accumulation in oil bodies remain unknown.

As molecular transport across biological phospholipid bilayers is generally mediated by membrane transporter proteins (Widhalm et al. 2015, Ichino and Yazaki 2022), one potential candidate for mediating SM transport to the oil body lumen is MpABCG1, which specifically localizes to the oil body membrane (Kanazawa et al. 2020). ATP-binding cassette (ABC) transporters, present in all kingdoms, mediate the transport of various molecules, including metals, hormones, lipids, and phenolics. ABC transporters consist of either a single nucleotide-binding domain (NBD) fused with a single set of transmembrane domains (TMDs), referred to as “half” transporters, or two NBDs and two sets of TMDs, referred to as “full” transporters. ATP hydrolysis in NBDs provides the driving force for substrate translocation across membranes, whereas TMDs form a tunnel through which the substrates pass (Wilkens 2015). Phylogenetic analyses have revealed that ABC transporters in the plant lineage are classified into eight subfamilies: A–G and I (Verrier et al. 2008). Specifically, the ABCG subfamily is markedly expanded in plants. Half ABCG transporters in plants are sometimes referred to as “white–brown complexes” because of their phylogenetic relationship with those involved in eye pigmentation in fruit flies (Gräfe and Schmitt 2021). For example, volatile terpenoids from petunia flowers are transported from the interior of petal cells to the atmosphere by a half ABCG transporter (Adebesin et al. 2017). Plants also possess a large group of full ABCG transporters that are absent in animals (Verrier et al. 2008). They form a distinct clade corresponding to the pleiotropic drug resistance subfamily and play various roles, including pathogen defense, diffusion barrier formation, and phytohormone transport, in plants (Gräfe and Schmitt 2021). Full ABCG transporters also participate in the deposition of cutin molecules and the secretion of antifungal terpenes and alkaloids (Ichino and Yazaki 2022). Additionally, both half and full ABCG transporters are involved in monoterpene emission from the flowers of scented orchid species (Chang et al. 2023).

In this study, we focused on the oil body-localized transporters, MpABCG1 and MpABCG36, which are closely related and show similar expression patterns in oil body cells. These two ABC transporters cooperatively contribute to the biosynthesis and accumulation of sesquiterpenes, with MpABCG1 playing a predominant role in the biosynthesis and accumulation of marchantins. Our findings provide new insights into the mechanisms underlying SM accumulation in the oil bodies of *M. polymorpha*.

## Results

### MpABCG1 and MpABCG36 are specifically expressed in oil body cells and localized to the oil body membrane in *M. polymorpha*

**Figure 1:**
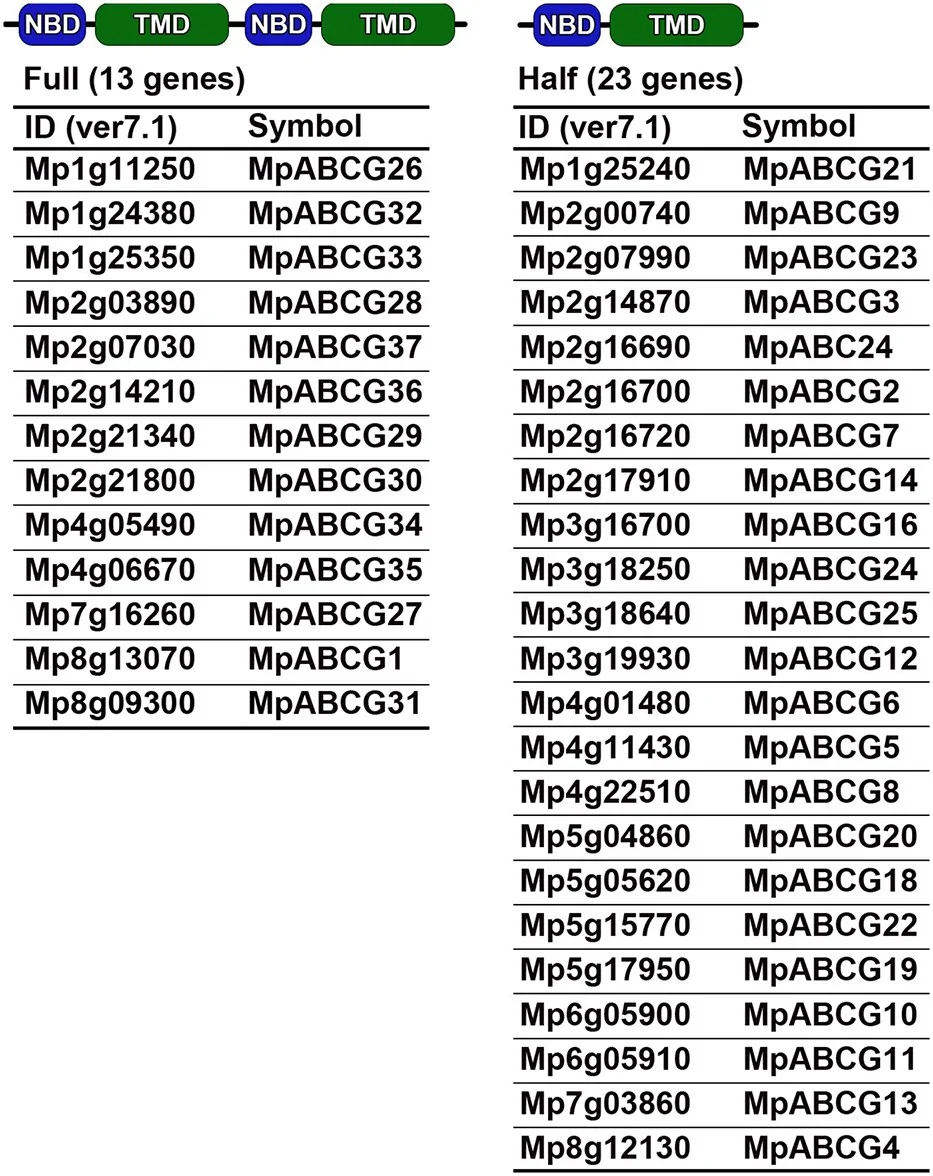
ABCG proteins in *M. polymorpha*. Full transporters consist of two NBDs and two sets of TMDs, whereas half transporters consist of only one NBD and a single set of TMDs. In total, 13 and 23 genes were annotated in the *Marchantia* genome database. Gene IDs and symbols are based on MarpolBase (https://marchantia.info/) (Tanizawa et al. 2026).

**Figure 2:**
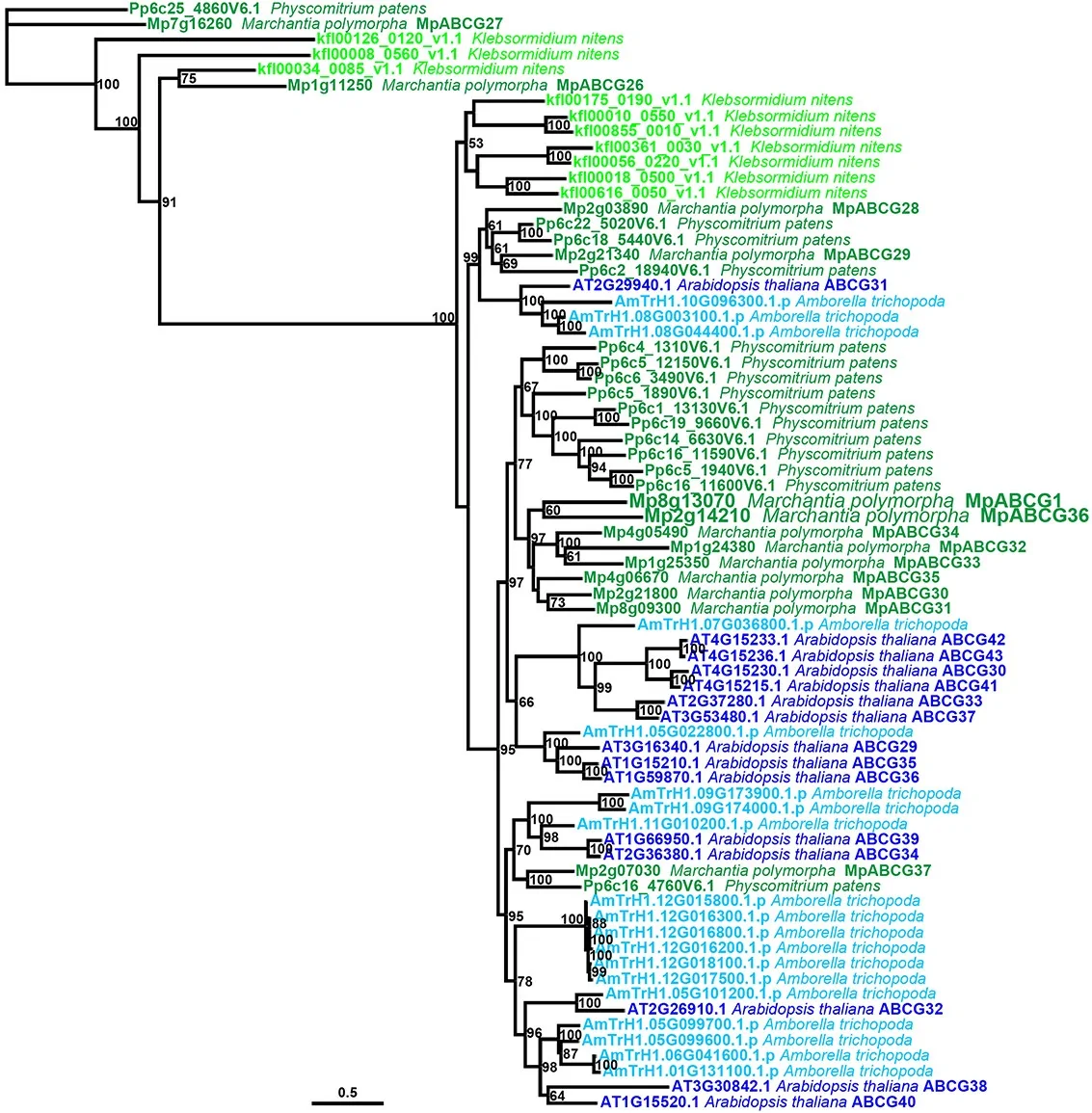
Maximum-likelihood phylogenetic tree of ABCG proteins in plants. Species names are indicated directly in the figure, including the charophyte green alga *Klebsormidium nitens*, the bryophytes *Marchantia polymorpha* and *Physcomitrium patens*, the basal angiosperm *Amborella trichopoda*, and the eudicot *Arabidopsis thaliana*. The branch length is proportional to the estimated number of substitutions per site. Bootstrap probabilities are indicated as percentages on branches with at least 50% support. A more detailed tree was previously constructed by Bowman et al. (2017).

To investigate the mechanisms underlying the accumulation of sesquiterpenoids and marchantins in the oil bodies of *M. polymorpha*, we focused on MpABCG1, which localizes to the oil body membrane (Kanazawa et al. 2020). MpABCG1 is composed of two NBDs and two sets of TMDs, arranged in the order NBD–TMD–NBD–TMD, thus representing a full ABCG protein (Fig. 1). *M. polymorpha* genome encodes 13 full and 23 half ABCG proteins (NBD–TMD; Fig. 1) (Bowman et al. 2017). To clarify the phylogenetic relationships among full ABCG proteins, we conducted a maximum-likelihood phylogenetic analysis and found that MpABCG36 (Mp2g14210) was phylogenetically close to MpABCG1 (Fig. 2).

**Figure 3:**
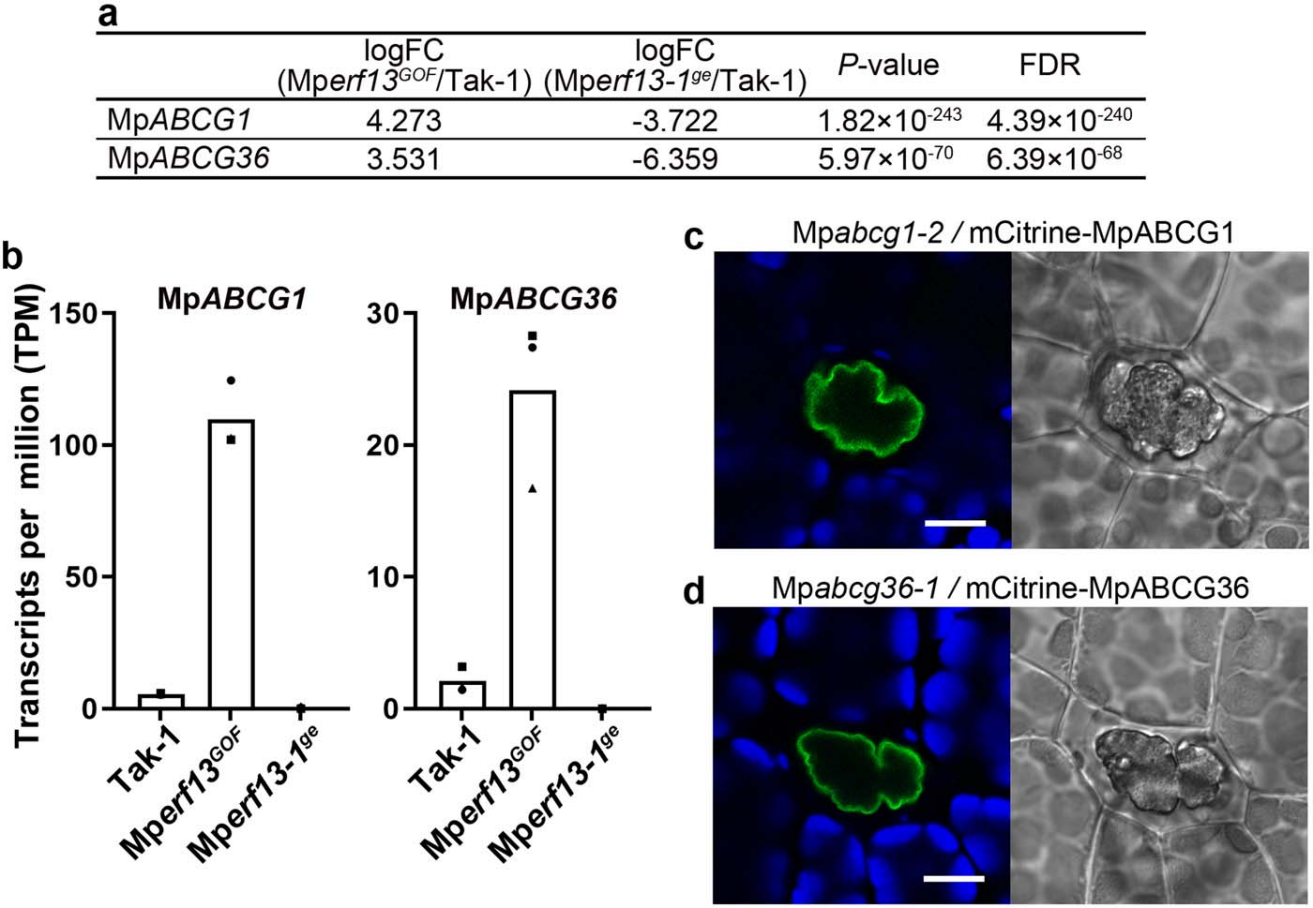
Expression levels of Mp*ABCG1* and Mp*ABCG36* in *M. polymorpha*. (a) Re-analysis of the RNA-sequencing data of Mp*erf13* mutants (Kanazawa et al. 2020). Both Mp*ABCG1* and Mp*ABCG36* exhibited a FDR < 0.01 and absolute logFC > 2. (b) Transcripts per million values of Mp*ABCG1* and Mp*ABCG36* re-calculated from the RNA-sequencing data (Kanazawa et al. 2020). (c and d) Single confocal images of thallus cells expressing mCitrine–MpABCG1 in the Mpabcg1-2 mutant (c) and mCitrine–MpABCG36 in the Mp*abcg36-1* mutant (d) under their native regulatory elements. Green and blue pseudo-colors indicate fluorescence from mCitrine and chlorophyll, respectively. Bright field images are also shown. Bars, 10 μm.

**Figure 4:**
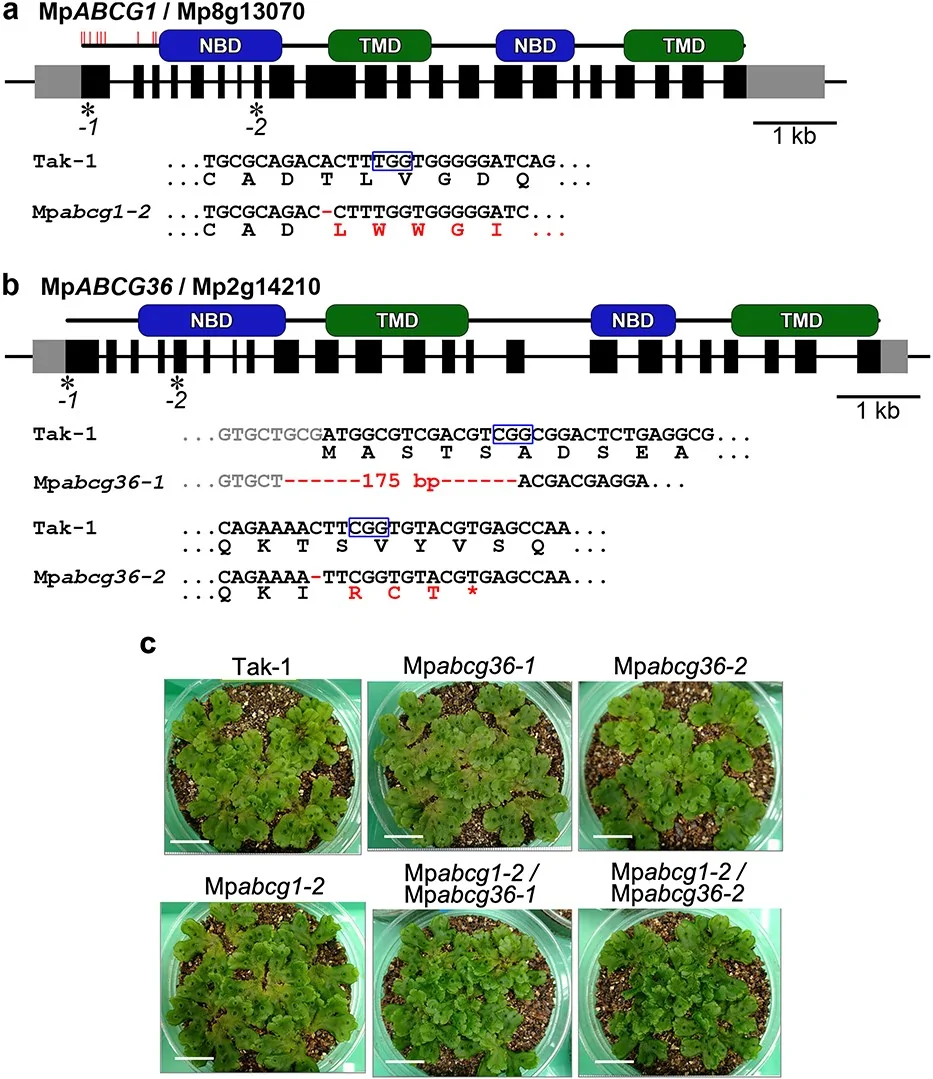
Mp*abcg1* and Mp*abcg36* mutants generated by CRISPR-based genome editing. (a and b) Gene structures of Mp*ABCG1* (a) and Mp*ABCG36* (b), with schematic representations of their protein domain organization (including NBD and TMD shown above. Gray and black boxes represent the untranslated (UTRs) and coding regions, respectively. Asterisks with numbers indicate the mutation sites. PAM and mutated sequences are boxed in blue and highlighted in red, respectively. Vertical lines indicate the positions of methionine residues located upstream of the functional domains, which could potentially serve as alternative translation initiation sites. The mutations in each line were verified by sequencing of both genomic DNA and cDNA. (c) Six-week-old thalli (2 weeks on half-strength B5 agar medium, followed by 4 weeks on vermiculite) of Tak-1 and Mp*abcg1*, Mp*abcg36*, and Mp*abcg1*/Mp*abcg36* mutants. Genotypes are labeled on each image. Bars, 2 cm.

We further examined the expression profiles of ABCG genes using published RNA-sequencing datasets (Kanazawa et al. 2020). Mp*ERF13* is a key transcription factor essential for oil body biogenesis. The gain-of-function (Mp*erf13^GOF^*) and loss-of-function (Mp*erf13-1^ge^*) mutants produce excessive and no oil bodies, respectively. Through the comparison between the three groups using an analysis of variance (ANOVA)-like test, we identified Mp*ABCG1* and Mp*ABCG36* genes whose expressions were higher than 2 log-fold change in Mp*erf13^GOF^* compared with Tak-1 (wild-type male accession of *M. polymorpha* Takaragaike-1), and lower than a −2 log-fold change in Mp*erf13-1^ge^* than Tak-1 [false discovery rate (FDR) < 0.01] (Fig. 3a and b) (Kanazawa et al. 2020). None of the other *ABCG* genes were classified as differentially expressed according to the experimental criteria (Supplementary Figs. S1 and S2). Furthermore, mining of single-cell RNA-sequencing data from thallus tissues revealed that the transcripts of both Mp*ABCG1* and Mp*ABCG36* were predominantly enriched in cluster 18, which corresponded to the oil body cell cluster (Supplementary Figs. S1 and S2) (Wang et al. 2023). To confirm their tissue distribution and subcellular localization, we introduced mCitrine–MpABCG1 and mCitrine–MpABCG36 constructs under the control of their respective native regulatory elements, including 5′-and 3′-flanking sequences and introns into the respective mutant backgrounds (details are described later). Both fusion proteins were specifically expressed in oil body cells and localized to the oil body membrane (Fig. 3c and d), suggesting that Mp*ERF13* positively regulates the transcription of Mp*ABCG1* and Mp*ABCG36* and that these ABCG proteins function in oil body cells. Next, we generated Mp*abcg1* and Mp*abcg36* mutants *via* clustered regularly interspaced palindromic repeat (CRISPR)/Cas9-mediated genome editing and obtained Mp*abcg1*/Mp*abcg36* double mutants by crossing (Fig. 4). Mp*abcg1-1*, Mp*abcg1-2*, Mp*abcg36-1*, and Mp*abcg36-2* alleles contained deletions of 8, 1, 175, and 1 bp, respectively, suggesting the loss of function in all cases (Fig. 4a and b, Supplementary Fig. S3a). Notably, both single (Mp*abcg1-1*, Mp*abcg1-2*, Mp*abcg36-1*, and Mp*abcg36-2*) and double (Mp*abcg1-1*/Mp*abcg36-1*, Mp*abcg1-1*/Mp*abcg36-2*, Mp*abcg1-2*/Mp*abcg36-1*, and Mp*abcg1-2*/Mp*abcg36-2*) mutants exhibited no obvious differences in thallus development compared to the wild type under the tested experimental conditions (Fig. 4c, Supplementary Fig. S3b). Similarly, Mp*abcg1-2* expressing mCitrine–MpABCG1 or Mp*abcg36-1* expressing mCitrine–MpABCG36 also showed no obvious differences (Supplementary Fig. S3c). This phenotype is consistent with previous reports that oil body-deficient mutants (Mp*erf13^ge^*, Mp*c1hdz^ge^*, and Mp*myb02^ge^*) grow normally relative to the wild type (Kanazawa et al. 2020, Romani et al. 2020, Kubo et al. 2024).

### Mp*ABCG1* and Mp*ABCG36* mutations exert partially overlapping effects on the accumulation of oil body sesquiterpenoids

**Figure 5:**
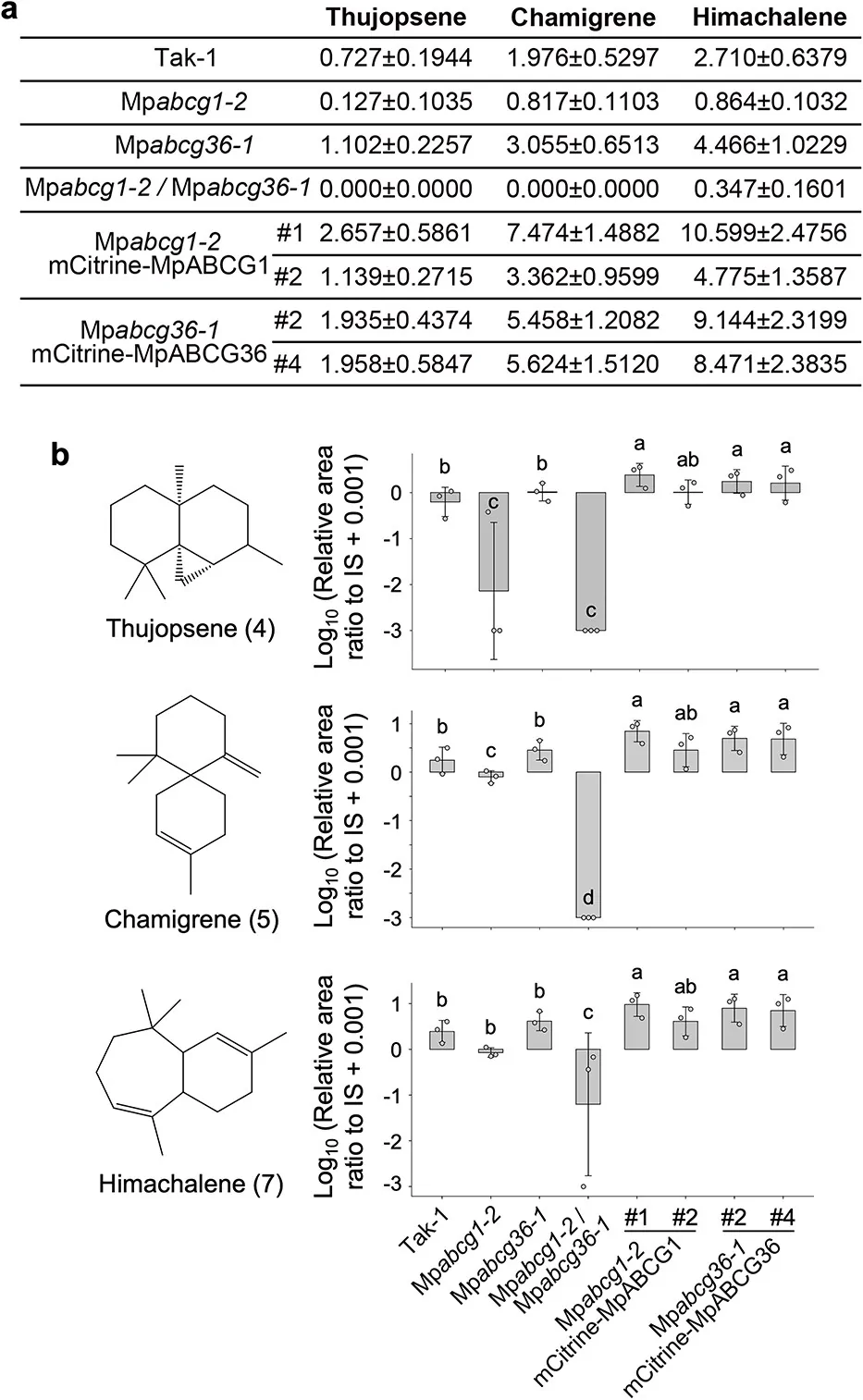
Oil body sesquiterpene levels in Mp*abcg1* and Mp*abcg36* mutants and their complemented lines. (a) Relative peak areas of each compound normalized to the IS (tetralin). Sesquiterpene levels were determined from extracts of 6-week-old thalli of Tak-1, Mp*abcg1-2*, Mp*abcg36-1*, and Mp*abcg1-2* / Mp*abcg36-1* thalli, as well as two independent complemented lines for each of Mp*abcg1-2* and Mp*abcg36-1*. Values represent mean ± standard error. (b) Structures and statistical analyses were performed on log10-transformed data. Bars represent mean ± SD (*n* = 3), with individual data points overlaid. Different letters indicate significant differences determined by one-way ANOVA followed by Tukey’s multiple comparison test (*P* < 0.05).

Previous microcapillary-based metabolite analysis of oil bodies has demonstrated that sesquiterpenoids, such as thujopsene, chamigrene, and himachalene, accumulate in the oil bodies of *M. polymorpha* (Tanaka et al. 2016). To examine the effects of Mp*ABCG1* and Mp*ABCG36* mutations on the accumulation of these sesquiterpenoids, we conducted gas chromatography–mass spectrometry (GC–MS) analyses (Fig. 5; Supplementary Figs. S4–S6). Among the single mutants, levels of sesquiterpenes in Mp*abcg1-1* were comparable to those in Tak-1, whereas a clear reduction was observed in Mp*abcg1-2*, which was restored by the introduction of mCitrine–MpABCG1 (see below), supporting that the phenotype is attributable to loss of MpABCG1 function rather than allele-specific effects. In contrast, both Mp*abcg36* alleles (Mp*abcg36-1* and Mp*abcg36-2*) showed similar phenotypes (Supplementary Fig. S6). For clarity, we present data from Mp*abcg1-2* and Mp*abcg36-1* as representative alleles in the main figures. Similar trends were observed for the accumulation of thujopsene and chamigrene (Fig. 5). Accumulation of thujopsene and chamigrene in Mp*abcg1-2* significantly decreased to 17.5 ± 14.2% and 41.3 ± 5.58% of those in Tak-1. In Mp*abcg36-1*, thujopsene and chamigrene levels showed an increasing trend compared with Tak-1; however, the difference was not statistically significant. Thujopsene and chamigrene were almost undetectable in the Mp*abcg1-2* / Mp*abcg36-1* double mutant (Fig. 5). The levels of himachalene tended to decrease to 36 ± 3.81% in Mp*abcg1-2* and tended to increase to 165 ± 37.7% in Mp*abcg36-1* compared with Tak-1; however, neither change was statistically significant. Nevertheless, in Mp*abcg1-2* / Mp*abcg36-1*, the level was significantly reduced to 12.9 ± 5.91% of that in Tak-1 (Fig. 5). The reduced levels observed in the mutants were restored to near wild-type levels by the introduction of mCitrine–MpABCG1 or mCitrine–MpABCG36, respectively (Fig. 5), indicating that these fusion proteins are functional and supporting that the observed phenotypes are specifically caused by the loss of MpABCG1 and MpABCG36 functions. These results collectively suggest that Mp*ABCG1* and Mp*ABCG36* play partially overlapping roles in mediating the accumulation of sesquiterpenoid compounds in the oil bodies of *M. polymorpha*.

### Mp*abcg1*, but not Mp*abcg36*, affects the accumulation of the bisbibenzyls, marchantins

**Figure 6:**
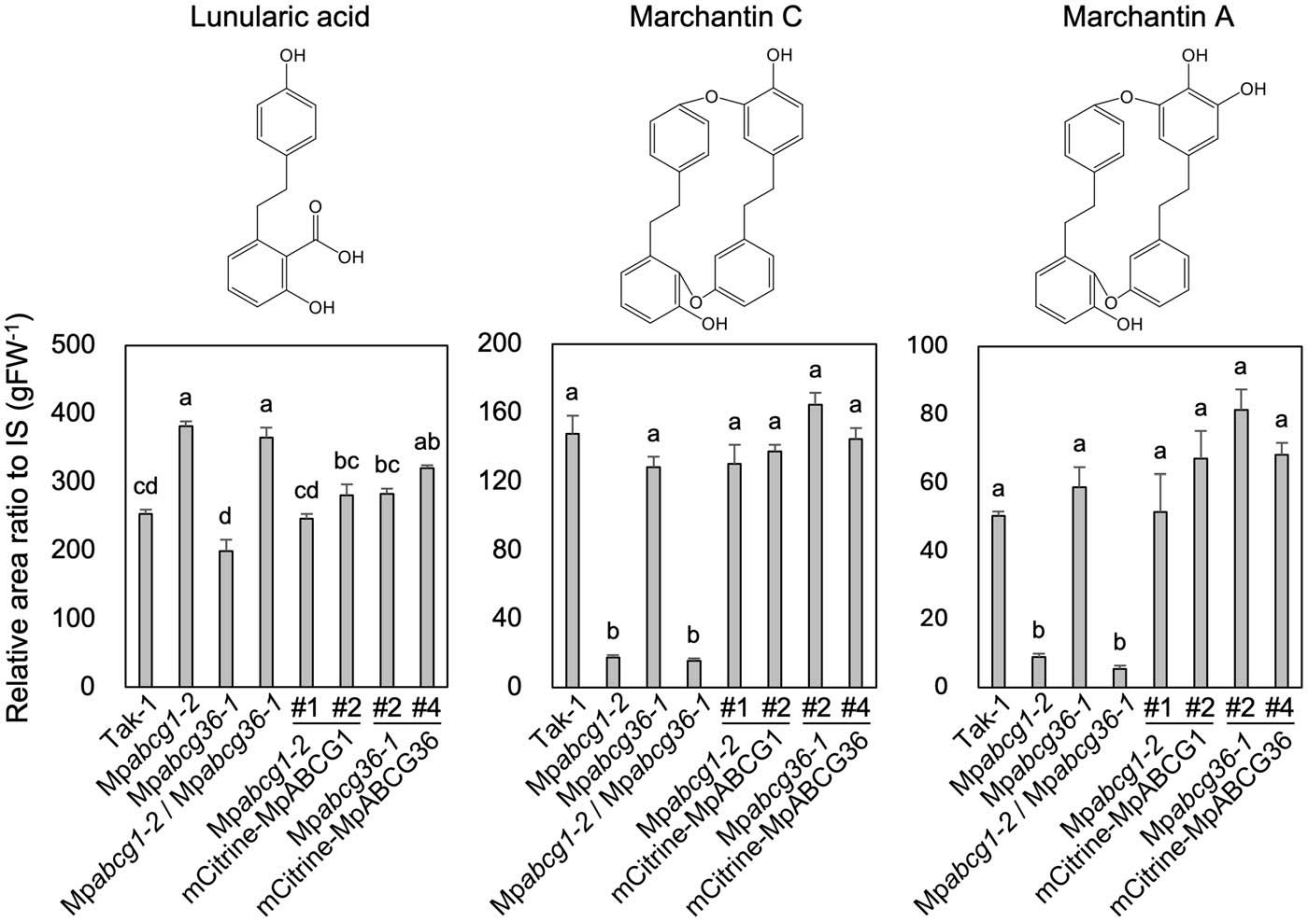
Predominant effect of the Mp*abcg1-2* mutation on marchantin accumulation. Structures and relative peak areas (mean ± SE; *n* = 3) of lunularic acid, marchantin C, and marchantin A normalized to IS. Different lowercase letters indicate the significant differences (one-way ANOVA, Tukey’s test; *P* < 0.05).

In addition to sesquiterpenoids, the oil bodies of *M. polymorpha* contain a characteristic bisbibenzyl compound, marchantin A, which is potentially biosynthesized from the bibenzyl precursor, lunularic acid (Friederich et al. 1999a, Tanaka et al. 2016). To quantify the accumulation of these compounds in the Mp*abcg1* and Mp*abcg36* mutants, we conducted liquid chromatography–tandem mass spectrometry (LC–MS/MS) analyses (Fig. 6; Supplementary Figs. S7 and S8). Accumulation levels of marchantin C were significantly reduced to 21.7 ± 3.18 and 19.5 ± 1.69% of those in Tak-1 in the Mp*abcg1-2* and Mp*abcg1-2* / Mp*abcg36-1* mutants, respectively, but remained comparable to those in the wild type in the Mp*abcg36-1* and Mp*abcg36-2* mutants (Fig. 6; Supplementary Fig. S8). Similar patterns were observed for marchantin A, whose accumulation levels significantly decreased to 13.7 ± 3.65 and 5.77 ± 1.38% in Mp*abcg1-2* and Mp*abcg1-2* / Mp*abcg36-1*, respectively, but were unaffected in Mp*abcg36-1* and Mp*abcg36-2* (Fig. 6; Supplementary Fig. S8). The decreased levels of marchantins A and C observed in Mp*abcg1-2* were restored to near wild-type levels by the introduction of mCitrine–MpABCG1 (Fig. 6). These results suggest that Mp*ABCG1*, but not Mp*ABCG36*, plays a predominant role in the accumulation of bisbibenzyl-type compounds, such as marchantins, in liverwort oil bodies.

No reduction in the levels of lunularic acid, which has not been previously detected in the oil bodies of *M. polymorpha* thalli (Tanaka et al. 2016), was observed in any of the mutant lines (Fig. 6; Supplementary Fig. S8).

### Oil bodies in the Mp*abcg1-2* mutant are almost devoid of SMs, as revealed by BODIPY 493/503 staining

**Figure 7:**
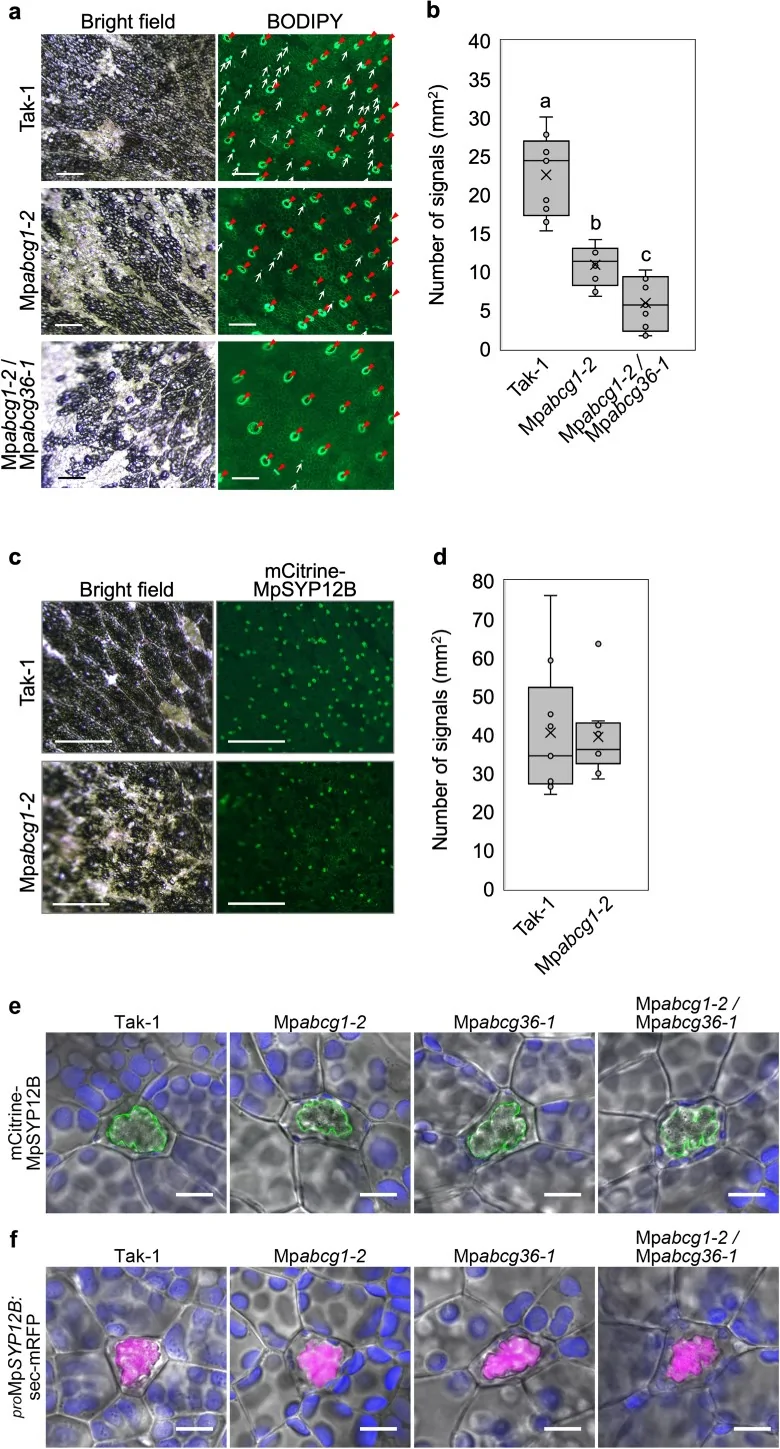
Effect of the Mp*abcg1-2* mutation on the number of BODIPY 493/503-stained oil bodies in *M. polymorpha* thalli. (a) Bright-field and fluorescent images of the BODIPY-stained thalli of Tak-1, Mp*abcg1-2*, and Mp*abcg1-2 / Mpabcg36-1*. Oil bodies are indicated by white arrows. Fluorescence from ring-like structures, indicated by arrowheads, represents air pores. Bars, 250 μm. (b) Number of oil bodies visualized by BODIPY per unit area (mm^2^; *n* = 9). Bars indicate the mean ± SE. Different lowercase letters indicate the significant differences (Tukey’s test; *P* < 0.05). (c) Bright-field and fluorescent images of the mCitrine–MpSYP12B-expressing thalli of Tak-1 and Mp*abcg1-2*. Arrows indicate oil bodies. Bars, 250 μm. (d) Number of oil bodies visualized by mCitrine–MpSYP12B per unit area (mm^2^). Bars indicate the mean ± SE. (e and f) Single confocal images of thallus cells expressing mCitrine–MpSYP12B (e) or sec-mRFP driven by the Mp*SYP12B* promoter (f) in Tak-1, Mp*abcg1-2*, Mp*abcg36-1*, and Mp*abcg1-2*/Mp*abcg36-1* mutants. Green, magenta, and blue pseudo-colors indicate fluorescence from mCitrine, mRFP, and chlorophyll, respectively. Bright field images are overlayed. Bars, 10 μm.

Accumulation of sesquiterpenoids and marchantins was reduced in the Mp*abcg1* and Mp*abcg36* mutants (Figs. 5 and 6), and their physiological effects were subsequently analyzed. BODIPY 493/503 (hereafter BODIPY), which exhibits a high affinity for lipophilic compounds, is a powerful tool to detect and visualize the oil bodies of *M. polymorpha* (Kanazawa et al. 2020, 2022, Gutsche et al. 2024). However, BODIPY does not exclusively stain oil bodies and can also label other lipophilic structures, such as lipid droplets and air pore cells. To avoid misinterpretation, oil bodies were manually identified and counted based on their characteristic morphology and localization within oil body cells. The numbers of BODIPY-stained oil bodies per unit area of thalli were 48.3 ± 3.88 and 26.3 ± 5.05 in Mp*abcg1-2* and Mp*abcg1-2* / Mp*abcg36-1*, respectively, with both showing a statistically significant decrease compared to that in Tak-1 (Fig. 7a and b).

We also examined the oil body membrane marker, mCitrine-MpSYP12B, in Tak-1 and Mp*abcg1-2 via* fluorescence stereomicroscopy. mCitrine-MpSYP12B predominantly localizes to the limiting membrane of the oil bodies of *M. polymorpha* (Kanazawa et al. 2020). Fluorescence signals of mCitrine-MpSYP12B were detected in both the wild-type and Mp*abcg1-2* plants (Fig. 7c). The number of mCitrine-MpSYP12B-labeled oil bodies per unit area of thalli showed no significant difference (Fig. 7d). To further confirm the presence of oil bodies, we performed confocal laser scanning microscopy using mCitrine–MpSYP12B and sec-mRFP driven by the Mp*SYP12B* promoter (an oil body lumen marker) in each mutant background. In all mutants, oil body membranes were labeled by mCitrine–MpSYP12B and the lumen was labeled by sec-mRFP, as observed in the Tak-1 background (Fig. 7e and f), indicating that oil bodies were normally formed in both the wild-type and Mp*abcg1* mutants. However, the Mp*abcg* mutation affected the accumulation of secondary metabolites in the oil bodies.

## Discussion

**Figure 8:**
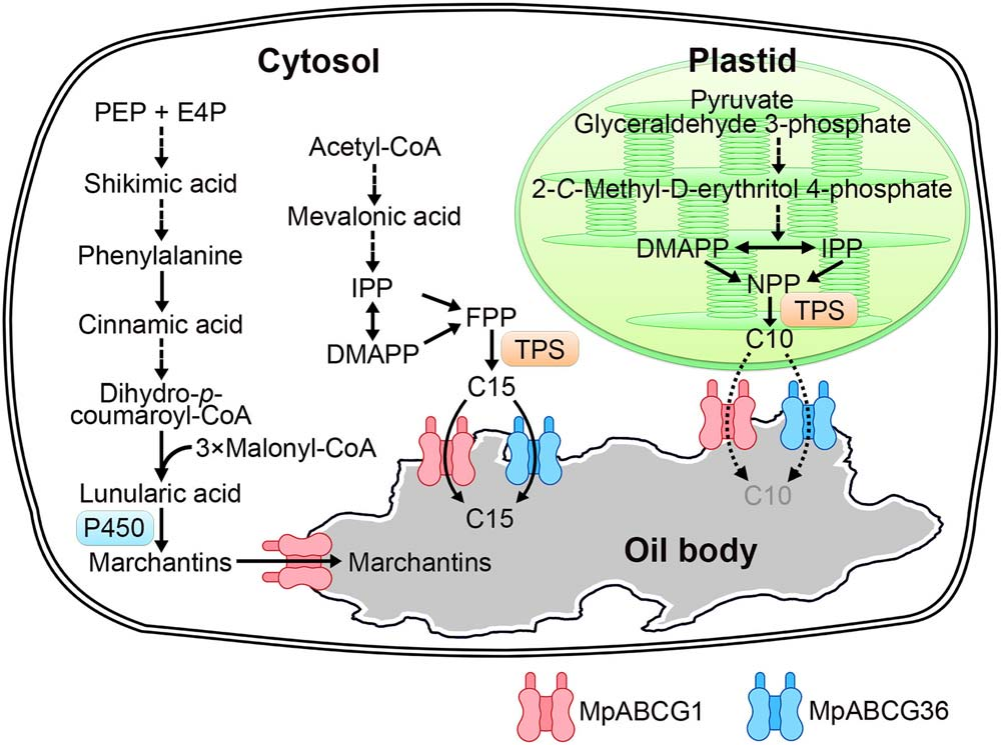
Proposed model of sesquiterpenoid and marchantin accumulation in the oil bodies of *M. polymorpha*. Schematic biosynthetic pathways of sesquiterpenes (C15), marchantins (bisbibenzyls), and monoterpenes (C10) are illustrated primarily based on the existing knowledge on angiosperms, as the corresponding pathways in *M. polymorpha* have not yet been fully elucidated. Both mCitrine–MpABCG1 and mCitrine–MpABCG36 localize specifically to the oil body membrane. MpABCG1 and MpABCG36 play overlapping roles in sesquiterpene accumulation in oil bodies, whereas MpABCG1 plays a predominant role in marchantin accumulation. Although Mp*abcg1*/Mp*abcg36* mutations affect the biosynthesis and/or accumulation of monoterpenes (C10), their oil body distribution remains unclear. PEP, phosphoenolpyruvic acid; E4P, erythrose 4-phosphate; P450, cytochrome P450; IPP, isopentenyl pyrophosphate; DMAPP, dimethylallyl pyrophosphate; NPP, neryl pyrophosphate; FPP, farnesyl pyrophosphate; TPS, terpene synthase; C10, monoterpenoids; C15, sesquiterpenoids.

In this study, we demonstrated that the ABCG proteins MpABCG1 and MpABCG36 are involved in the accumulation and potential transport of SMs and/or their precursors to liverwort oil bodies and proposed a model for SM accumulation in the oil bodies of *M. polymorpha* (Fig. 8). Liverworts produce and accumulate various SMs (He et al. 2013, Kanazawa et al. 2020, Romani et al. 2020). The oil bodies of *M. polymorpha* accumulate many SMs, including sesquiterpenoids (thujopsene, chamigrene, and himachalene) and the bisbibenzyl (marchantin A), as revealed *via* single-cell extraction using microcapillaries, followed by direct mass spectrometry analysis (Tanaka et al. 2016). However, the mechanisms underlying SM accumulation remain unclear.

This study first focused on MpABCG1, which was previously reported to localize to the oil body membrane in *M. polymorpha* (Kanazawa et al. 2020). Reanalysis of the RNA-sequencing data of Mp*erf13* mutants and confocal microscopic observation of fluorescent protein fusions revealed that both MpABCG1 and MpABCG36 function at the oil body membrane (Figs. 1–3). Levels of oil body sesquiterpenes (thujopsene and chamigrene) were decreased in the Mp*abcg1* mutant generated *via* CRISPR–Cas9-mediated genome editing. In the Mp*abcg36* single mutant, sesquiterpene levels did not differ substantially from those in the wild type. However, in the double mutant (Mp*abcg1-2* / Mp*abcg36-1*), the reduction in sesquiterpene levels observed in the Mp*abcg1* single mutant was more pronounced, particularly for chamigrene and himachalene. Moreover, the accumulation of bisbibenzyls (marchantin C and marchantin A) was reduced in Mp*abcg1,* but not in Mp*abcg36* (Figs. 4–6). Although SM accumulation was reduced, the number of mCitrine-MpSYP12B-labeled oil bodies in the Mp*abcg1-2* mutant was comparable to that in the wild type (Fig. 7), suggesting that MpABCG1 and MpABCG36 are essential for SM accumulation in oil bodies, but not for oil body formation. Together, our results support the view that liverwort oil bodies represent lineage-specific organelles specialized for the sequestration of hydrophobic bioactive metabolites, rather than simple lipid storage compartments.

Mp*ABCG1* was initially identified as a differentially expressed gene *via* RNA-sequencing analysis of Mp*erf13* mutants, and mCitrine-MpABCG1 was reported to be specifically expressed in oil body cells and localized to the oil body membrane (Kanazawa et al. 2020). In this study, we generated two mutant alleles, Mp*abcg1-1* and Mp*abcg1-2*, the latter of which showed the reduced accumulation of sesquiterpenes and marchantins. Considering the completely different biosynthetic pathways of sesquiterpenes and marchantins, MpABCG1 is possibly involved in the accumulation of diverse compounds. Although Mp*abcg1-1* was predicted to be a frameshift mutation introducing a premature stop codon, it did not affect the accumulation of sesquiterpenes and marchantins (Supplementary Figs. S6 and S8). One possible explanation is that translation may initiate from downstream methionine codons, potentially generating a truncated protein retaining partial activity; however, this remains to be experimentally tested (Supplementary Fig. S3a). Forestier et al. (2026) independently generated loss-of-function Mp*abcg1* mutant lines and demonstrated a marked reduction in sesquiterpene accumulation in these lines. Importantly, our conclusion that MpABCG1 contributes to the accumulation of sesquiterpenes and marchantins is supported independently by the phenotype of Mp*abcg1-2* and its complementation by mCitrine-MpABCG1 (Figs. 5 and 6). These findings suggest that MpABCG1 plays an essential role in sesquiterpene accumulation in oil bodies.

In this study, Mp*abcg1-2* / Mp*abcg36-1* mutant exhibited a reduced accumulation of three sesquiterpenes (thujopsene, chamigrene, and himachalene), monoterpenes (e.g. limonene), bisbibenzyls, and an oxylipin-related compound, 1-octen-3-yl acetate (Supplementary Figs. S4 and S5). This suggests that MpABCG1 and MpABCG36 play pleiotropic roles in the biosynthesis and accumulation of structurally diverse compounds. However, whether compounds other than sesquiterpenes and marchantins also accumulate in oil bodies remains unclear, warranting further investigation. Existing knowledge on angiosperms suggests that sesquiterpenoids, marchantins, and monoterpenes are biosynthesized in the cytosol or plastids (Fig. 8) (Vranová et al. 2013), through the pathways involving several biosynthetic enzymes and cofactors, such as NADPH and acetyl-CoA (Chapple 1998, Kihara et al. 2014). However, their functional subcellular distribution in *M. polymorpha* has not yet been fully characterized. An important limitation of the present study is that we did not directly demonstrate the transport activity of MpABCG1 and MpABCG36 toward the oil body SMs including sesquiterpenes or their precursors. Although our genetic, cell biological, and metabolite analyses strongly support their involvement in SM accumulation in oil bodies, the exact transported substrates and transport mechanism remain unresolved. Direct biochemical approaches, such as liposome-based transport assays, will be required to address this question in future studies.

Oil bodies contain marchantin A (Tanaka et al. 2016). Our study indicated that MpABCG1 was a major contributor to the accumulation of both marchantins C and A in oil bodies. P450 enzymes are necessary for the conversion of marchantin C to marchantin A (Friederich et al. 1999b), and most canonical P450 enzymes function at the ER (Chapple 1998); therefore, marchantins C and A are possibly biosynthesized on the cytosolic surface of the ER and subsequently transported to the lumen of oil bodies. As reported for volatile organic compounds (Widhalm et al. 2015), inhibition of marchantin sequestration may lead to the excessive accumulation of highly hydrophobic marchantins in the cytosol, thereby impairing the endomembrane integrity. In this study, marchantin levels were lower in the Mp*abcg1-2* mutant than those in the wild type, and plant growth appeared normal under the tested experimental conditions (Figs. 4c and 6). These findings suggest the presence of a feedback control system and/or a homeostatic mechanism that regulates the metabolic flux and marchantin accumulation. The lunularic acid level was significantly higher in Mp*abcg1-2* than in Tak-1, and similar metabolic profiles have been reported in Mp*myb02* (Kubo et al. 2018). Unlike Mp*abcg1-2*, Mp*myb02* undergoes oil body cell differentiation, but not oil body formation (Wang et al. 2023, Kubo et al. 2024). Comparative analyses of these mutants will provide deeper insights into the feedback control system and/or homeostatic mechanism preventing cellular toxicity caused by excessive marchantin accumulation. Importantly, our findings highlight the compartmentalization of SMs at high concentrations by organelles that have evolved in a lineage-specific manner.

## Materials and Methods

### Phylogenetic analysis

Protein sequences were collected from Phytozome 13 and the following genome portal sites: *Amborella trichopoda* v1.0, *Arabidopsis thaliana* TAIR11, *Klebsormidium nitens* NIES-2285 v1.1 (http://www.plantmorphogenesis.bio.titech.ac.jp/∼algae_genome_project/klebsormidium/klebsormidium_blast.html), *M. polymorpha* v5.1, and *Physcomitrium patens* v3.3. Multiple sequence alignments were performed using MUSCLE version 3.8.31 with default parameters (Edgar 2004a, 2004b). Maximum-likelihood phylogenetic analyses were conducted using PhyML 3.0 under LG model (Guindon et al. 2010). Bootstrap support values were estimated from 1000 replicates.

### RNA-sequencing re-analysis

RNA-sequencing data (accession no. DRA009193) were used for re-analysis (Kanazawa et al. 2020). Transcript abundance was quantified using kallisto (version 0.43.1) with default parameters and the primary transcript dataset from *Marchantia* genome version 3.1 (Bray et al. 2016, Bowman et al. 2017). Single-cell RNA-sequencing data were mined using Single Cell Browser (http://wanglab.sippe.ac.cn/Marchantia-census/) (Wang et al. 2023).

### DNA constructs

All oligonucleotides and DNA constructs used in this study are listed in Table 1. To construct mCitrine–MpABCG36, a 14.5-kb genomic fragment of Mp*ABCG36* containing the 5′- and 3′-flanking sequences was amplified by PCR from Tak-1 genomic DNA, and the amplified product was subcloned into pENTR/D-TOPO (Thermo Fisher Scientific, Waltham MA, USA), according to the manufacturer’s instructions. The coding sequence of mCitrine was subsequently inserted upstream of the initiation codon of Mp*ABCG36* using the In-Fusion HD Cloning Kit (Takara, Shiga, Japan), according to the manufacturer’s instructions. The resultant construct was introduced into the pMpGWB301 Gateway binary vector (Ishizaki et al. 2015) using the Gateway LR Clonase II Enzyme Mix (Thermo Fisher Scientific).

**Table 1:** List of primers and DNA constructs used in this study.

Purpose	Target gene	Primer (5′ to >3′)	Size (kb)
Genome edit	Mp*abcg1-1*	ctcgAAGTGGGAGCATGAGGGATT	-
		aaacAATCCCTCATGCTCCCACTT	
	Mp*abcg1-2*	ctcgGGATATTTGCGCAGACACTT	-
		aaacAAGTGTCTGCGCAAATATCC	
	Mp*abcg36-1*	ctcgTGCTGCGATGGCGTCGACGT	-
		aaacACGTCGACGCCATCGCAGCA	
	Mp*abcg36-2*	ctcgGTTCGTTGCACAGAAAACTT	-
		aaacAAGTTTTCTGTGCAACGAAC	
Entry	CDS for Mp*ABCG36*	caccactagtggcggcagcggcATGGCGTCGACGTccgCGGACTCTGAGG	10.8 kb
		CATTGCGTAGCATTAAGCGAGCATACAG	
	*_pro_* Mp*ABCG36*	gcaggctccgcggccCAGAGGCTATGTCGGCTCAGAAGCTTG	3.7 kb
		gtgaagggggcggccCGCAGCACTCTCGCAGAGTCGCGTCC	
	*mCitrine*	CCCCTTCACCACTAGATGGTGAGCAAGGGCGAGGAG	0.7 kb
		CGCTGCCGCCACTAGCCTTGTACAGCTCGTCCATGCC	
**DNA constructs**		**Source**	**Identifier**
pMpGWB301 Genomic mCitrine-MpSYP12B		Kanazawa et al. 2016	n.a.
pMpGWB301 Genomic mCitrine-MpABCG1		Kanazawa et al. 2020	n.a.
pMpGWB301 Genomic mCitrine-MpABCG36		This study	n.a.
pMpGWB301 *_pro_*Mp*SYP12B:*sec-mRFP		Kanazawa et al. 2020	n.a.
pMpGE_En03		Sugano et al. 2018	LC090755
pMpGE_En03 Mp*abcg1-1*		This study	−
pMpGE_En03 Mp*abcg1-2*		This study	−
pMpGE_En03 Mp*abcg36-1*		This study	−
pMpGE_En03 Mp*abcg36-2*		This study	−
pMpGE011		Sugano et al. 2018	LC090757
pMpGE011 Mp*abcg1-1*		This study	−
pMpGE011 Mp*abcg1-2*		This study	−
pMpGE011 Mp*abcg36-1*		This study	−
pMpGE011 Mp*abcg36-2*		This study	−

To construct genome-editing vectors, double-stranded DNA fragments were inserted into the BsaI site of pMpGE_En03 (Sugano et al. 2018). The resultant constructs were introduced into the pMpGE011 Gateway binary vector (Sugano et al. 2018) using the Gateway LR Clonase II Enzyme Mix, following the manufacturer’s instructions.

### Plant growth and transformation

All strains used in this study are listed in Table 2. Male and female accessions of *M. polymorpha* Takaragaike-1 (Tak-1) and Takaragaike-2 (Tak-2), respectively, were used for analyses (Ishizaki et al. 2008). Growth conditions and transformation methods were as previously described (Kubota et al. 2013, Kanazawa et al. 2020, Takizawa et al. 2021). Gemmae were aseptically cultured on half-strength Gamborg’s B5 medium (Fujifilm Wako Pure Chemicals, Osaka, Japan) containing 1% agar in plastic Petri dishes (diameter: 90 mm; depth: 20 mm; Sansei Medical, Kyoto, Japan) at 22°C under continuous fluorescent light (25–61 μmol photons m^−2^ s^−1^). After cultivation on agar plates for 2 weeks, the thalli were transplanted onto vermiculite (thickness: 3.5–4.0 cm) soaked in 2000-fold diluted Hyponex nutrient solution (Hyponex Japan, Osaka, Japan). The thalli were further cultured on vermiculite at 22°C under continuous fluorescent light in a growth chamber. The vermiculite was kept moist *via* regular watering, and no additional nutrients were supplied after initial application of the diluted Hyponex solution. Far-red light was supplemented to induce the transition from vegetative to reproductive growth. Mutants generated *via* genome editing were crossed with the wild-type accessions to remove the Cas9 cassette.

**Table 2:** List of strains used in this study.

Experimental models: *M. polymorpha* strains	Source
*M. polymorpha* Tak-1	Ishizaki et al. 2008
*M. polymorpha* Tak-2	Ishizaki et al. 2008
*M. polymorpha* mCitrine-MpSYP12B	Kanazawa et al. 2016
*Marchantia polymorpha _pro_*Mp*SYP12B:*sec-mRFP	Kanazawa et al. 2020
*M. polymorpha* Mp*abcg1-1^ge^*	This study
*M. polymorpha* Mp*abcg1-2^ge^*	This study
*M. polymorpha* Mp*abcg36-1^ge^*	This study
*M. polymorpha* Mp*abcg36-2^ge^*	This study
*M. polymorpha* Mp*abcg1-1^ge^* / Mp*abcg36-1^ge^*	This study
*M. polymorpha* Mp*abcg1-1^ge^* / Mp*abcg36-2^ge^*	This study
*M. polymorpha* Mp*abcg1-2^ge^* / Mp*abcg36-1^ge^*	This study
*M. polymorpha* Mp*abcg1-2^ge^* / Mp*abcg36-2^ge^*	This study
*M. polymorpha* Mp*abcg1-2^ge^* / mCitrine-MpABCG1	This study
*M. polymorpha* Mp*abcg36-1^ge^* / mCitrine-MpABCG36	This study
*M. polymorpha* Mp*abcg1-2^ge^* / mCitrine-MpSYP12B	This study
*M. polymorpha* Mp*abcg36-1^ge^* / mCitrine-MpSYP12B	This study
*M. polymorpha* Mp*abcg1-2^ge^* / Mp*abcg36-1^ge^* / mCitrine-MpSYP12B	This study
*M. polymorpha* Mp*abcg1-2^ge^* / *_pro_*Mp*SYP12B:*sec-mRFP	This study
*M. polymorpha* Mp*abcg36-1^ge^* / *_pro_*Mp*SYP12B:*sec-mRFP	This study
*M. polymorpha* Mp*abcg1-2^ge^* / Mp*abcg36-1^ge^* / *_pro_*Mp*SYP12B:*sec-mRFP	This study

### GC–MS analysis of volatile compounds

Thalli grown for 4 weeks on vermiculite after transplanting from agar plates were used for volatile analysis, as previously described (Takizawa et al. 2021). The thalli (~100 mg fresh weight) were placed in a 2-mL screw-cap tube (Sarstedt, Nümbrecht, Germany) and snap-frozen in liquid nitrogen with eight stainless steel beads (diameter: 3 mm). After the addition of 0.4 mL methyl *tert*-butyl ether (Fujifilm Wako Pure Chemicals, Osaka, Japan) containing 2.0 μg mL^−1^ tetralin as an internal standard (IS), the thalli were vigorously lysed using a bead cell disrupter (Micro Smash MS-100R; Tomy, Tokyo, Japan) at 3500 rpm for 1 min. The mixture was centrifuged at 15 000 rpm for 3 min at 25°C, and the upper organic phase was directly subjected to GC–MS (Tanaka et al. 2016). A GC–MS system (QP-5050; Shimadzu, Kyoto, Japan) equipped with the DB-WAX column (length × inner diameter: 30 m × 0.25 mm; film thickness: 0.25 μm; Restek, Bellefonte, PA, USA) was used. The column temperature was set as follows: 40°C for 5 min, increased to 200°C at 5°C min^−1^, and held for 5 min. Helium was used as the carrier gas at a flow rate of 40 cm s^−1^. Injector and interface temperatures were set at 200°C. Splitless injections were performed with a sampling time of 1 min. The mass spectrometer was operated in the electron impact mode with an ionization energy of 70 eV. The relative area ratio of each peak to that of tetralin (IS) was used to quantify each volatile compound. Thujopsene, chamigrene, and himachalene were identified by comparing their MS spectra and retention times with those of authentic standards kindly provided by Dr H. Kenmoku and Dr Y. Asakawa (Tokushima Bunri University, Tokushima, Japan). Limonene and 1-octen-3-yl acetate were purchased from Fujifilm Wako Pure Chemicals. Other compounds were tentatively identified by comparing their MS spectra with those in the National Institute of Standards and Technology-17 database (Shimadzu).

### LC–MS/MS analysis of marchantins

Marchantin levels were estimated as previously described (Tanaka et al. 2016). Apical portion of the thalli containing the primordia (~10 mg) was excised with a razor blade, placed in a 2.0-mL screw-cap microtube with seven stainless steel beads (diameter: 3 mm), snap-frozen in liquid nitrogen, and stored at −80°C until use. To each sample, 400 μL methanol containing 20.0 μg mL^−1^ nordihydroguaiaretic acid (Fujifilm Wako Pure Chemicals) as IS was added, followed by two rounds of homogenization at 3500 rpm for 1 min using a bead-type cell disruptor (Micro Smash MS-100R; Tomy). After centrifugation at 15 000 × g for 3 min, the supernatant was directly analyzed using the 3200 QTRAP system (AB Sciex, Framingham, MA, USA) equipped with the Prominence UFLC system (Shimadzu).

Marchantins were separated on the Mightysil RP-18 column (150 mm × 2 mm; 5 μm; Kanto Chemical, Tokyo, Japan) with a binary solvent system consisting of H₂O/HCOOH (100:0.1 [v/v]; solvent A) and CH₃CN/HCOOH (100:0.1 [v/v]; solvent B). The elution program consisted of a linear gradient from 20% solvent B (5 min) to 90% solvent B for 35 min at a flow rate of 0.2 mL min^−1^. Detection was performed using a photodiode array detector (SPD-M20A; Shimadzu) and MS/MS with electrospray ionization in the positive-ion mode [ion spray voltage: 3000 V; temperature: 120°C; N₂ as curtain gas (12 arbitrary units) and collision gas set to “high”; collision energy: 6 V; declustering potential: 30 V]. The relative amounts of marchantins were calculated from the ratio of the analyte peak area to that of IS.

### Stereomicroscopy

Apical portion of the thalli (1 cm) was excised using a razor blade and examined under a fluorescence stereomicroscope (AF6000 Macro; Leica, Wetzlar, Germany). A GFP3 filter set (excitation: 470/40 nm; emission: 525/50 nm) was used to detect BODIPY and mCitrine fluorescence. For BODIPY staining, the excised thallus piece was immersed in 1 mL of 3.82 μM BODIPY solution in darkness for 15 min.

### Confocal laser-scanning microscopy

Five-day-old thalli were used for observations. The samples were mounted in half-strength Gamborg’s B5 liquid medium and observed using an LSM780 confocal microscope (Carl Zeiss, Germany) equipped with an oil immersion lens (63×, numerical aperture = 1.4) and lambda detectors. Excitation was performed at 488 nm (argon laser) and 561 nm (DPSS 561-10), and emissions were collected between 482 and 659 nm. Spectral unmixing of the obtained images was performed using the ZEN 2.3 SP1 software (Carl Zeiss). Representative confocal microscopy images of at least 20 biological replicates are shown.

## Supplementary Material

Supplementary_figs_S1-S8__pcag069

## Data Availability

All data supporting the findings of this study are included in the manuscript and the online supplementary material of this article. The data underlying this article will be shared on reasonable request to the corresponding author.
