# Supplementary material for: ABCG transporters are involved in the accumulation of specialized metabolites in the oil bodies of Marchantia polymorpha
Source: Plant Cell Physiol. 2026 May 23;67(8):1442–54. doi: 10.1093/pcp/pcag069 (PMC13545645; doi:10.1093/pcp/pcag069)
Supplement: Supplementary file 1 [file supplementary_figs_s1-s8__pcag069.pdf]

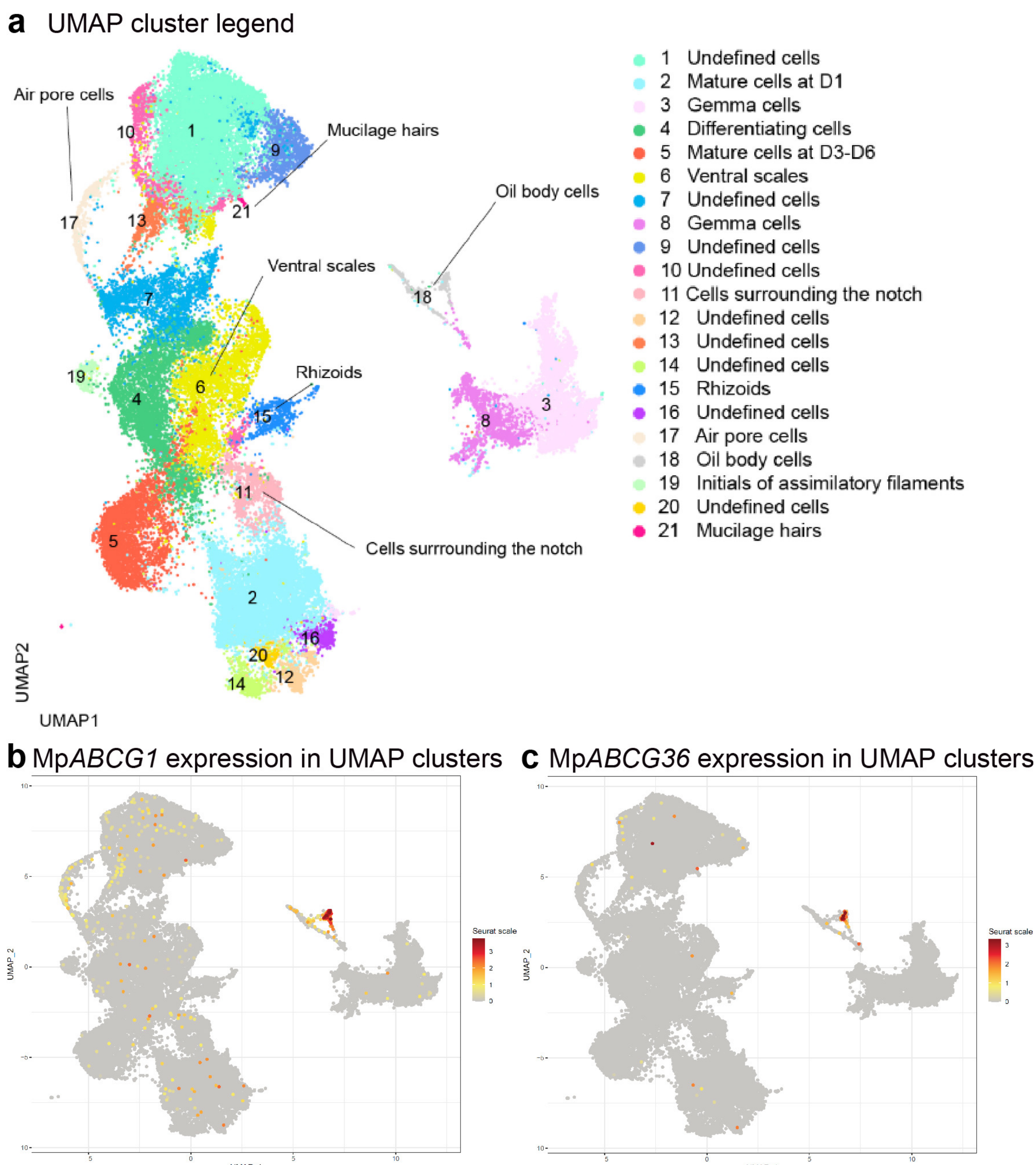

Supplementary Figure S1. Transcriptional expression profiles of Mp*ABCG1* and Mp*ABCG36*.

(a) Uniform manifold approximation and projection (UMAP) visualization of the cell clusters in *M. polymorpha* reported by Wang et al. (2023). **(b)** Mp*ABCG1* expression levels in UMAP clusters. **(c)** Mp*ABCG36* expression levels in UMAP clusters. Both Mp*ABCG1* and Mp*ABCG36* were predominantly plotted in cluster 18 (oil body cell cluster). UMAPs were generated using http://wanglab.sippe.ac.cn/Marchantia-census/ (Wang et al. 2023).

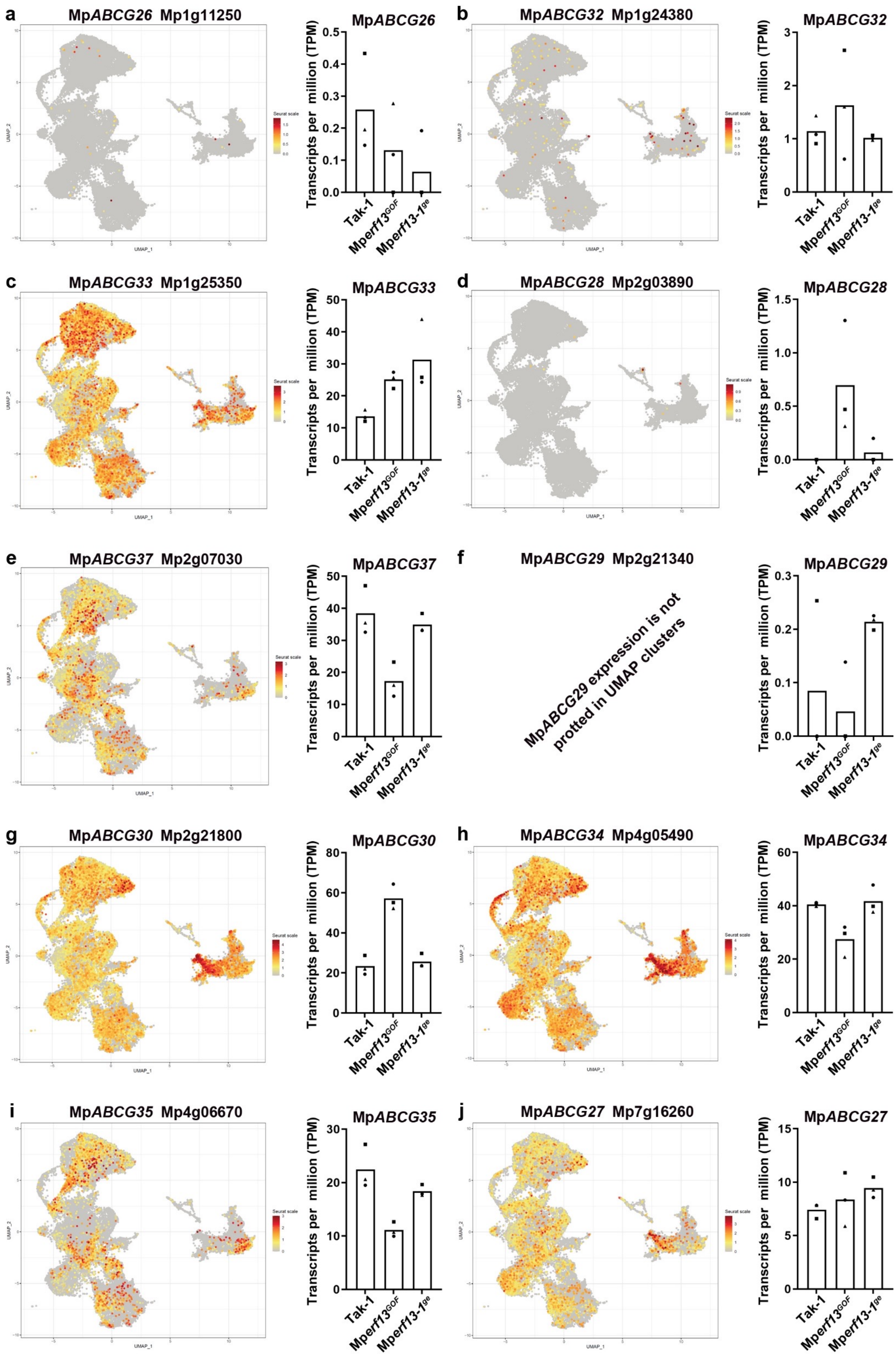

Supplementary Figure S2. Transcriptional expression profiles of all *ABCG* genes in *M. polymorpha*. Expression levels of all *ABCG* genes are shown as UMAP cluster distributions generated using Single Cell Browser (Wang et al., 2023) and transcripts per million (TPM) values re-calculated from the RNA-sequencing data of Kanazawa et al. (2020). Data for Mp*ABCG1* and Mp*ABCG36* are presented in Figure 3 and Supplementary Figure S1, respectively. Full and half *ABCG* genes are presented in panels **(a–k)** and **(l–ah),** respectively.

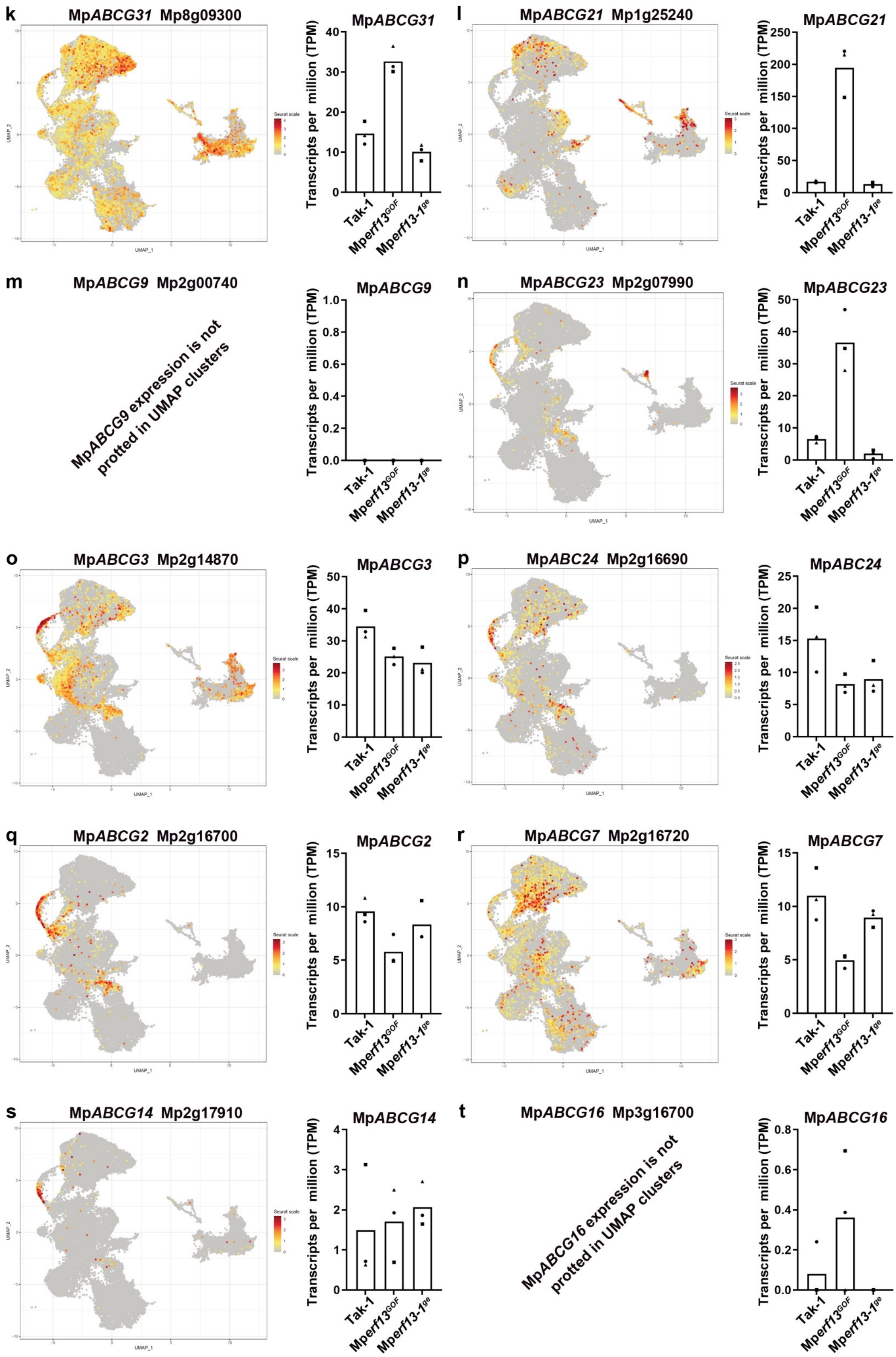

Supplementary Figure S2. (Continued).

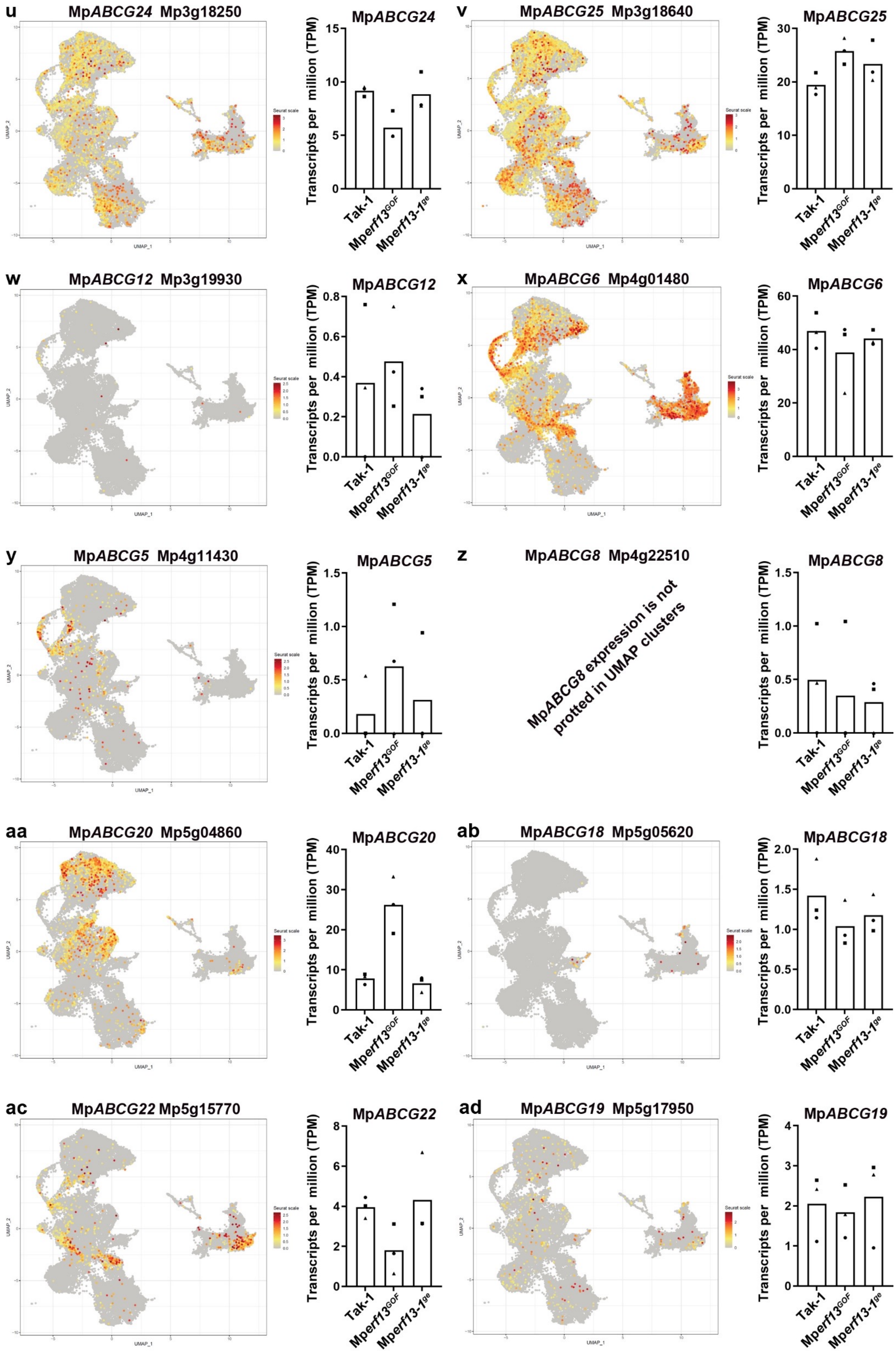

Supplementary Figure S2. (Continued).

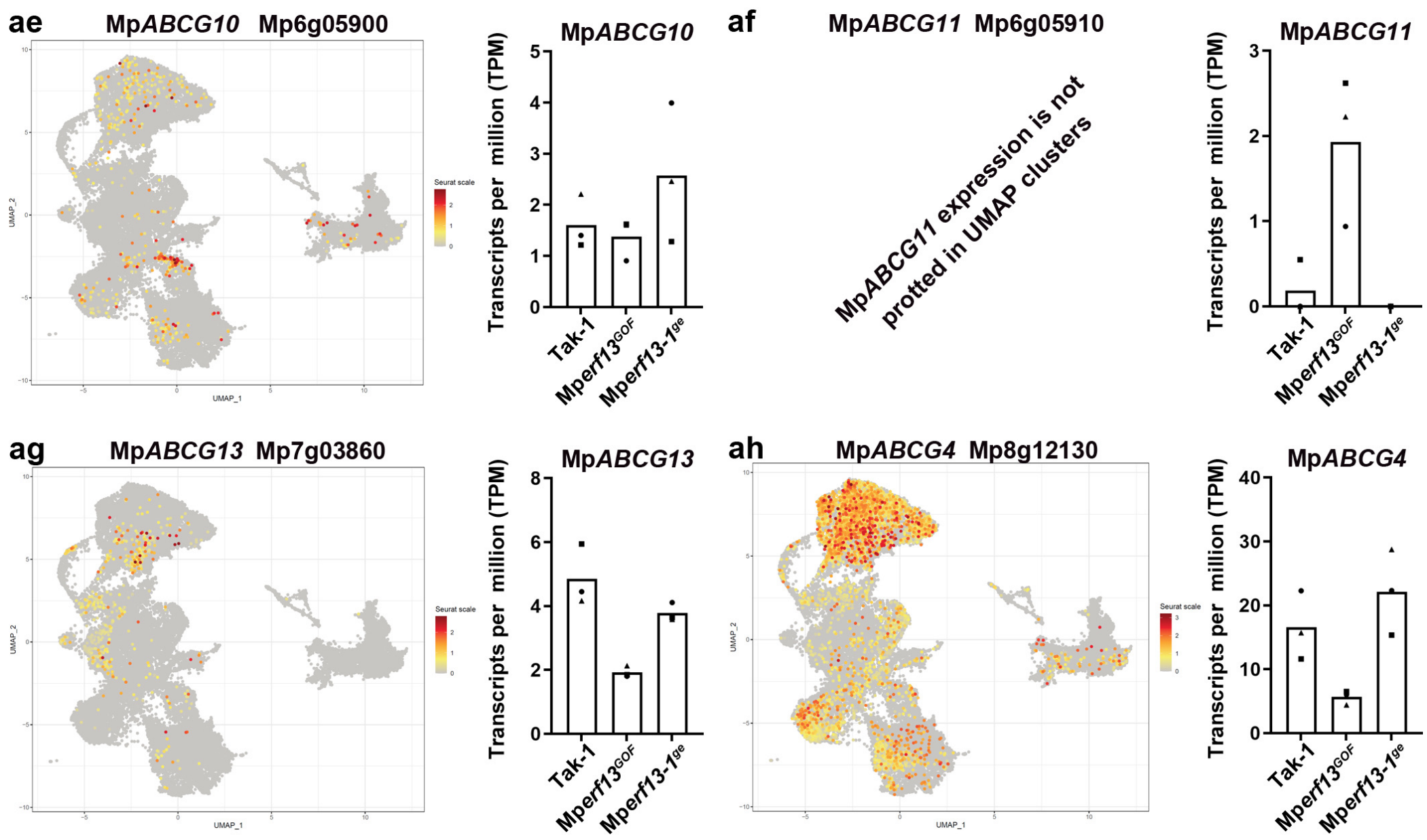

Supplementary Figure S2. (Continued).

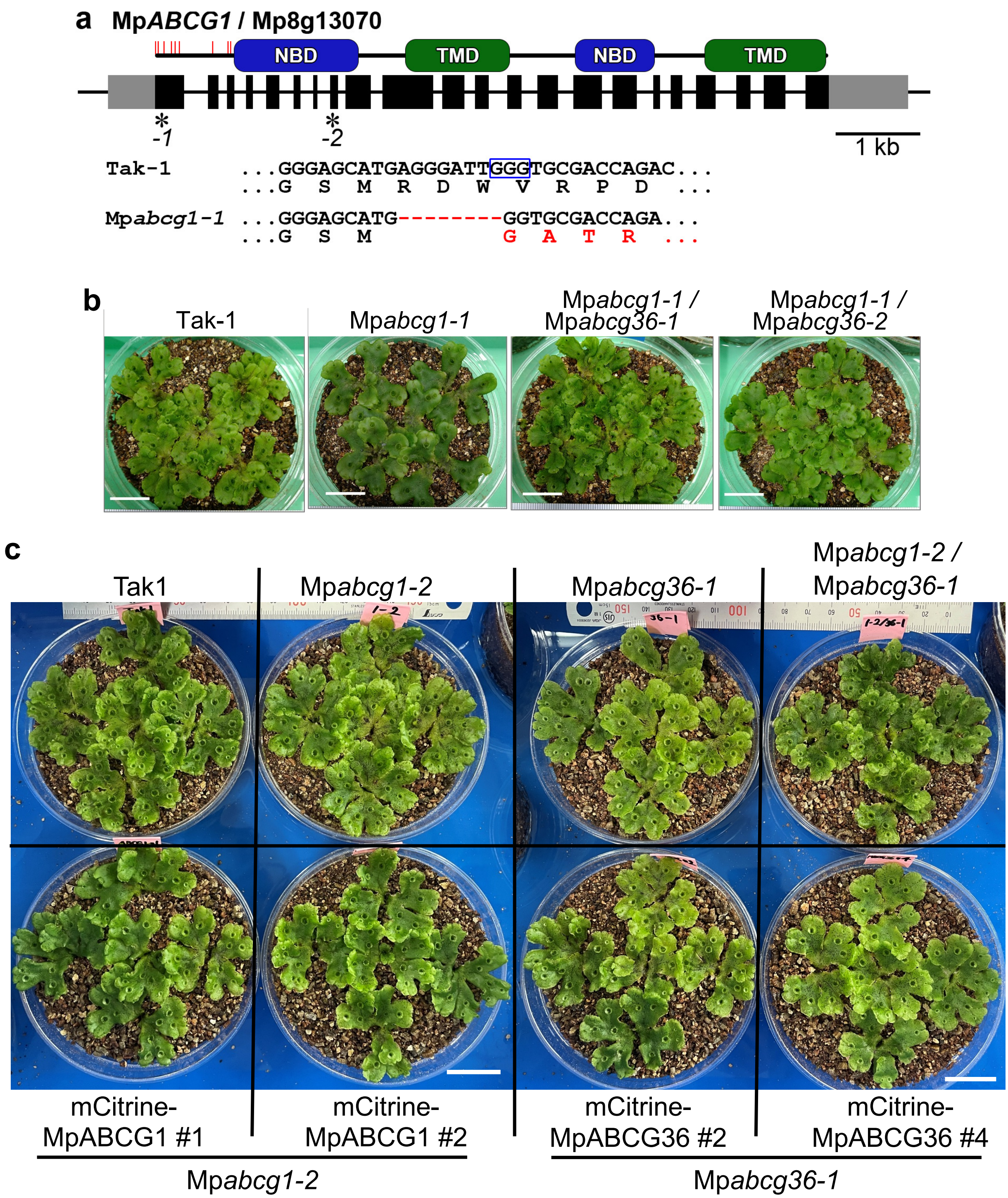

Supplementary Figure S3. Development of *Marchantia* thallus.
(a) Gene structure of Mp*ABCG1* and a schematic representation of its protein domain organization for the independent mutant allele Mp*abcg1-1*, in addition to the representative allele Mp*abcg1-2* shown in Figure 4. **(b and c)** Six-week-old thalli (cultured for two weeks on half-strength Gamborg's B5 agar medium, followed by four weeks on vermiculite) of Tak-1, Mp*abcg1-1*, Mp*abcg1-1* / Mp*abcg36-1*, and Mp*abcg1-1* / Mp*abcg36-2* **(b)**, or Tak-1, Mp*abcg1-2*, Mp*abcg36-1*, and Mp*abcg1-2* / Mp*abcg36-1*, as well as two independent lines of mCitrine–MpABCG1 / Mp*abcg1-2* and mCitrine–MpABCG36 / Mp*abcg36-1* **(c)**, are shown. Genotypes are indicated on each image. Bars, 2 cm.

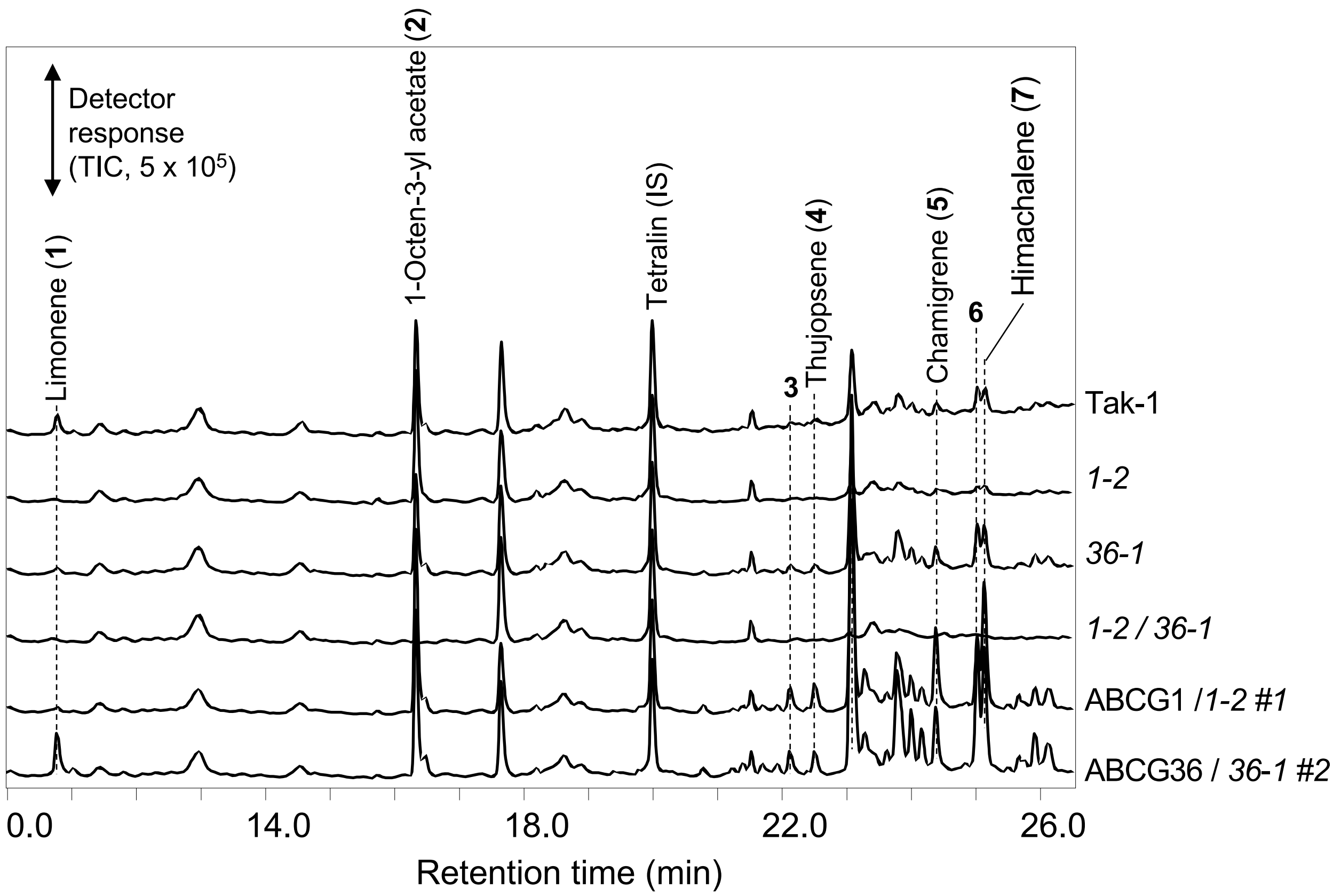

Supplementary Figure S4. Representative chromatograms obtained by gas chromatography–mass spectrometry (GC–MS) analysis of extracts from *M. polymorpha* thalli.

Chromatograms of Tak-1, Mp*abcg1-2* (*1-2*), Mp*abcg36-1* (*36-1*), and Mp*abcg1-2*/Mp*abcg36-1* (*1-2 / 36-1*) thalli, as well as those complemented lines of Mp*abcg1-2* and Mp*abcg36-1* expressing mCitrine–MpABCG1 (ABCG1 / *1-2* #1) and mCitrine–MpABCG36 (ABCG36 / *36-1* #2), respectively, are shown. Thalli were cultured for two weeks on half-strength Gamborg's B5 agar plates, followed by four weeks on vermiculite. Mass spectra of peaks 1–7 are shown in Supplementary Figure S5. Peaks identified using authentic standards are labeled with the corresponding compound names.

<u>1. D-Limonene (94)</u>

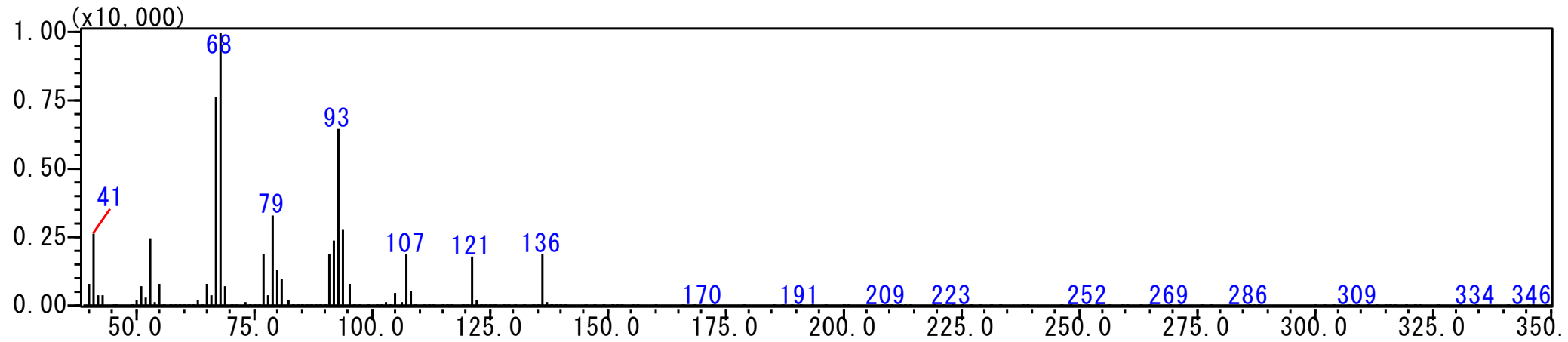

<u>2. 1-Octen-3-yl-acetate (94)</u>

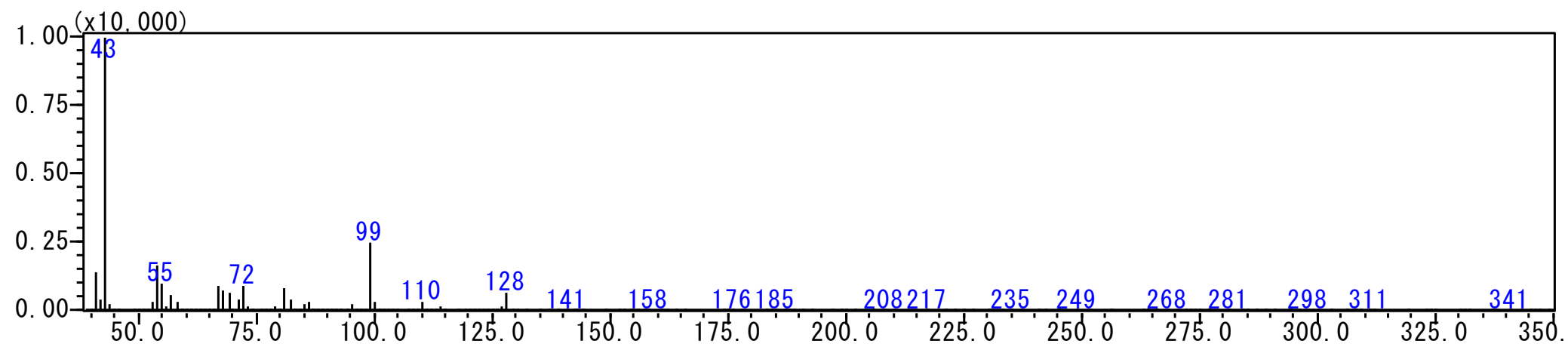

3. α-Chamigrene (89)

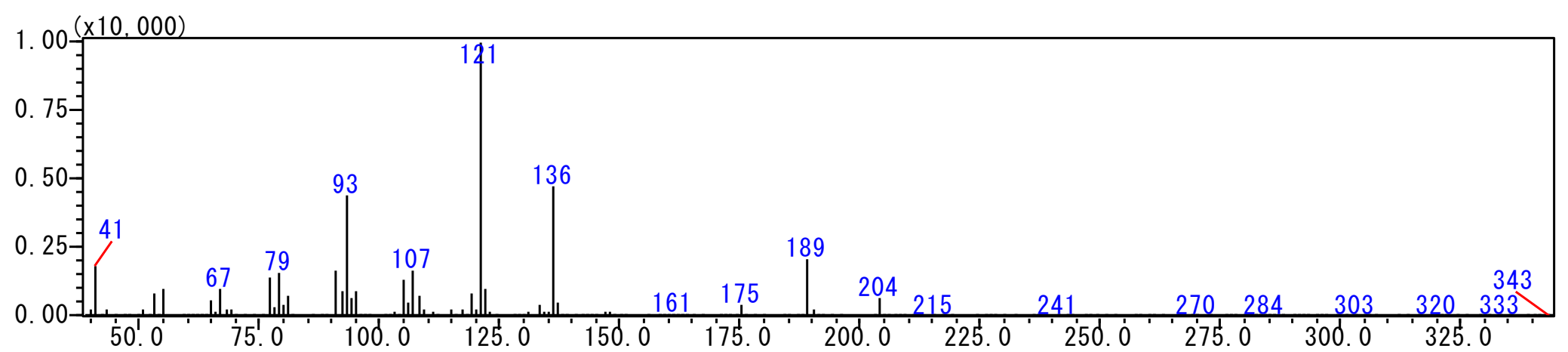

<u>4. Thujopsene (96)</u>

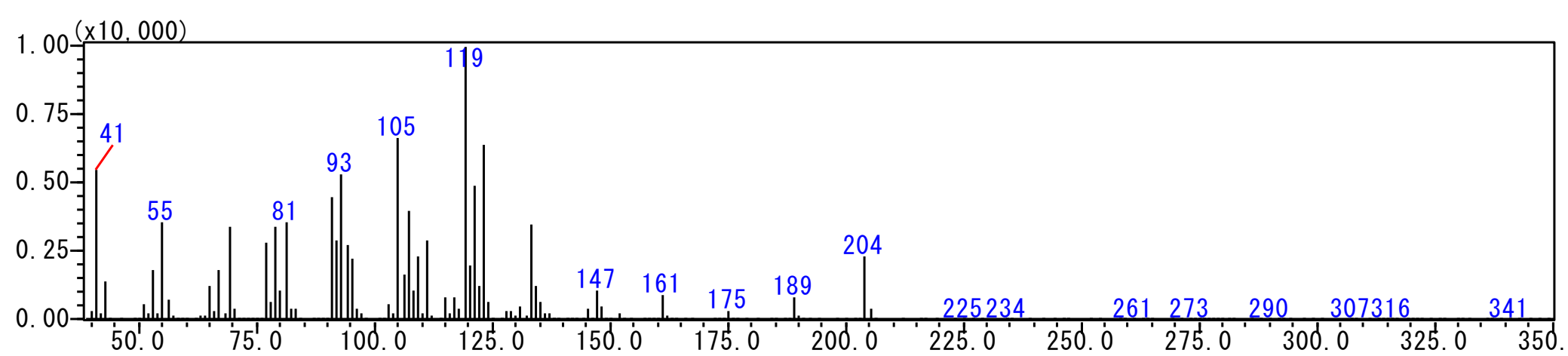

Supplementary Figure S5. Mass spectra of compounds 1–7.
For each compound, the most similar match in the National Institute of Standards and Technology (NIST)-17 library is shown, with the similarity score indicated in parentheses. Compounds with underlined names were identified using authentic standards.

5. β-Chamigrene (95)

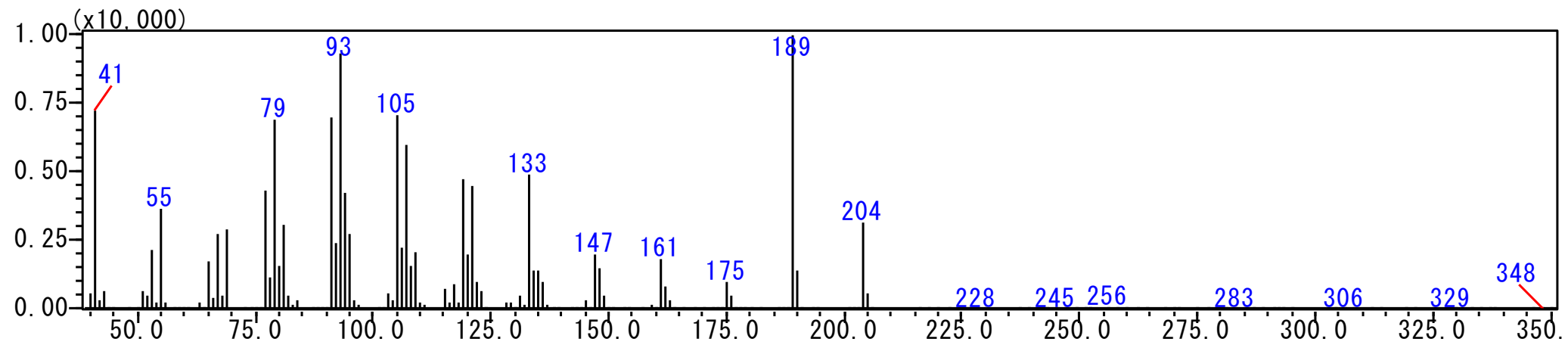

6. *cis*-α-Bisabolen (92)

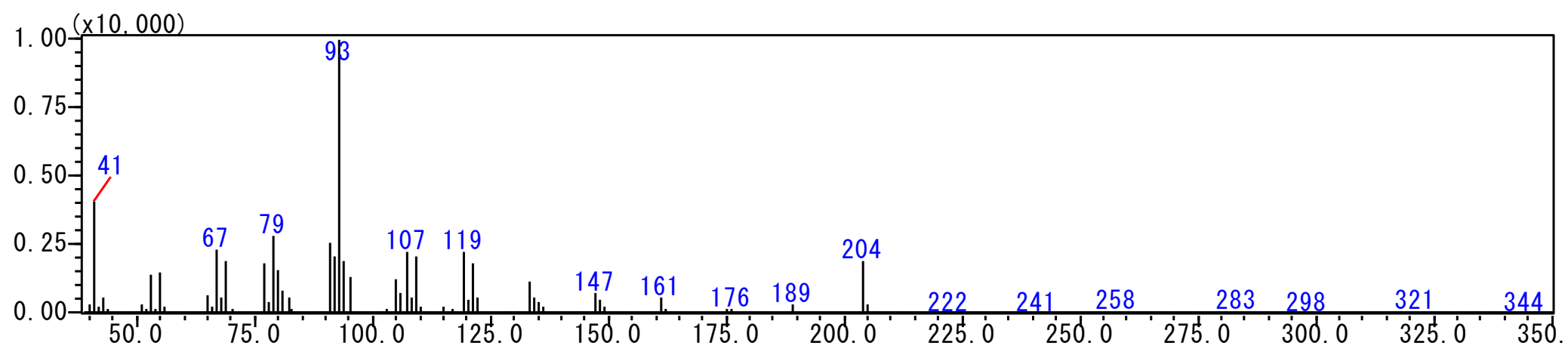

7. β-Himachalene (95)

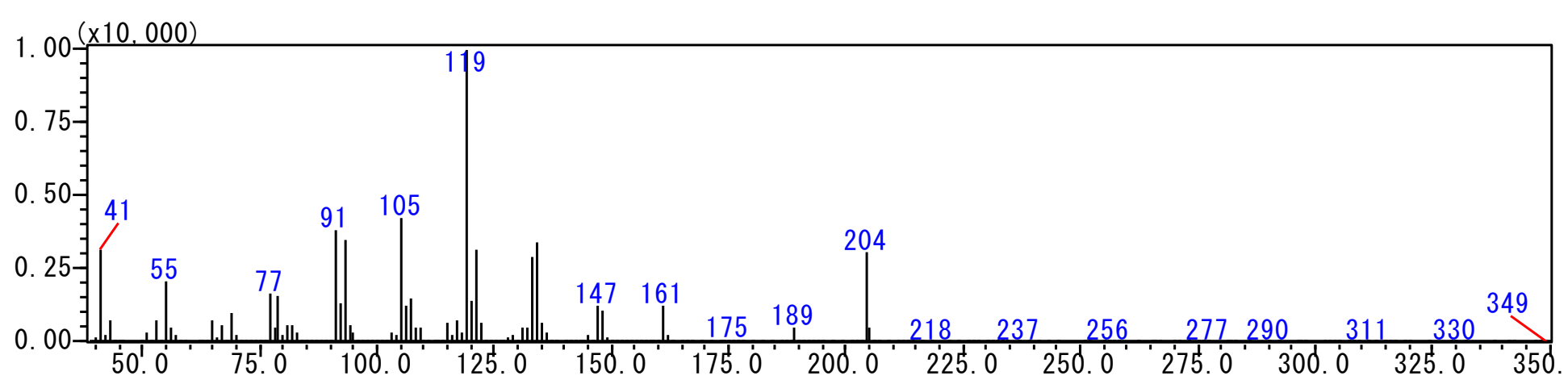

Supplementary Figure S5. (Continued).

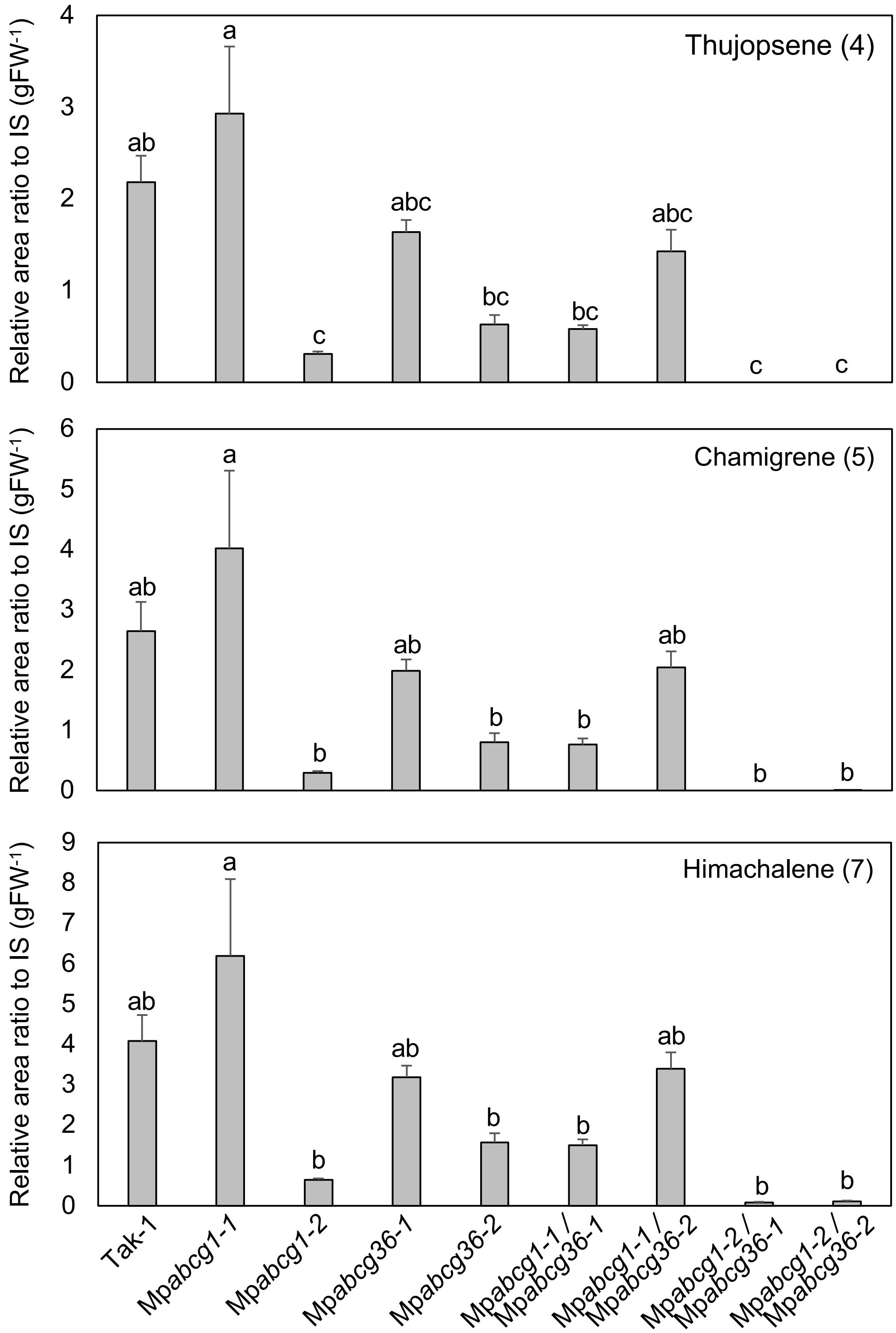

Supplementary Figure S6. Levels of sesquiterpenoids in all mutants generated in this study. Levels of three sesquiterpenoids identified in the extracts of the six-week-old thalli of all mutants generated in this study are shown. The relative peak area of each compound normalized to the internal standard (IS, tetralin) is shown. Data are represented the mean ± SE of three biological replicates. Different lowercase letters indicate the significant differences (one-way analysis of variance [ANOVA], followed by Tukey's test; $P < 0.05$).

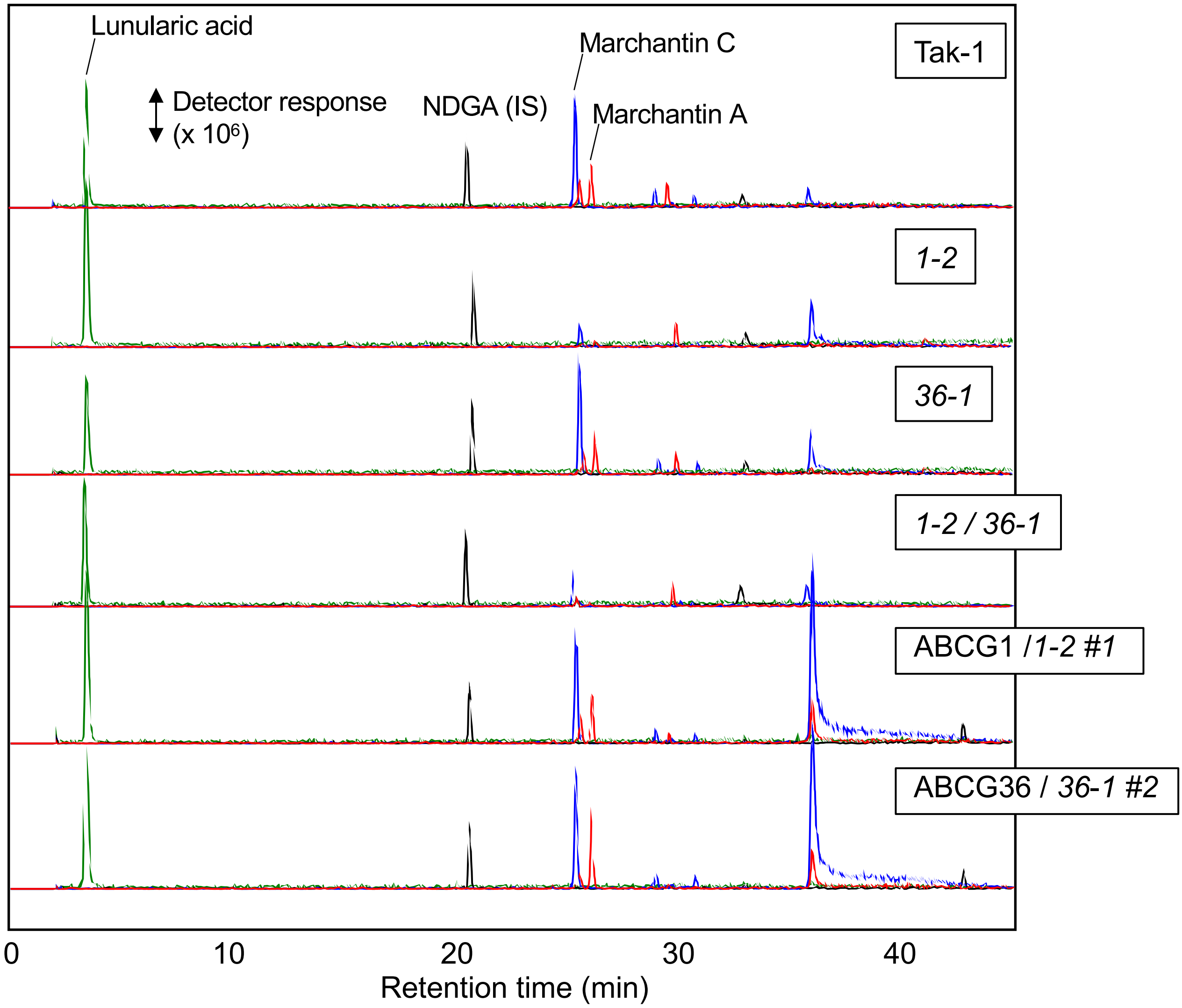

Supplementary Figure S7. Representative chromatograms obtained by liquid chromatography (LC)–MS/MS analysis of extracts from *M. polymorpha* thalli. Chromatograms of Tak-1, Mp*abcg1-2* (*1-2*), Mp*abcg36-1* (*36-1*), and Mp*abcg1-2*/Mp*abcg36-1* (*1-2 / 36-1*) thalli, as well as those complemented lines of Mp*abcg1-2* and Mp*abcg36-1* expressing mCitrine–MpABCG1 (ABCG1 / *1-2* #1) and mCitrine–MpABCG36 (ABCG36 / *36-1* #2), respectively, are shown. Thalli were cultured for two weeks on half-strength Gamborg's B5 agar plates, followed by four weeks on vermiculite. Extracted ion chromatograms correspond to lunularic acid (*m/z* 258.1 ± 0.5; green), nordihydroguaiaretic acid (NDGA; internal standard; *m/z* 302.2 ± 0.5; black), marchantin C (*m/z* 424.2 ± 0.5; blue), and marchantin A (*m/z* 440.2 ± 0.5; red).

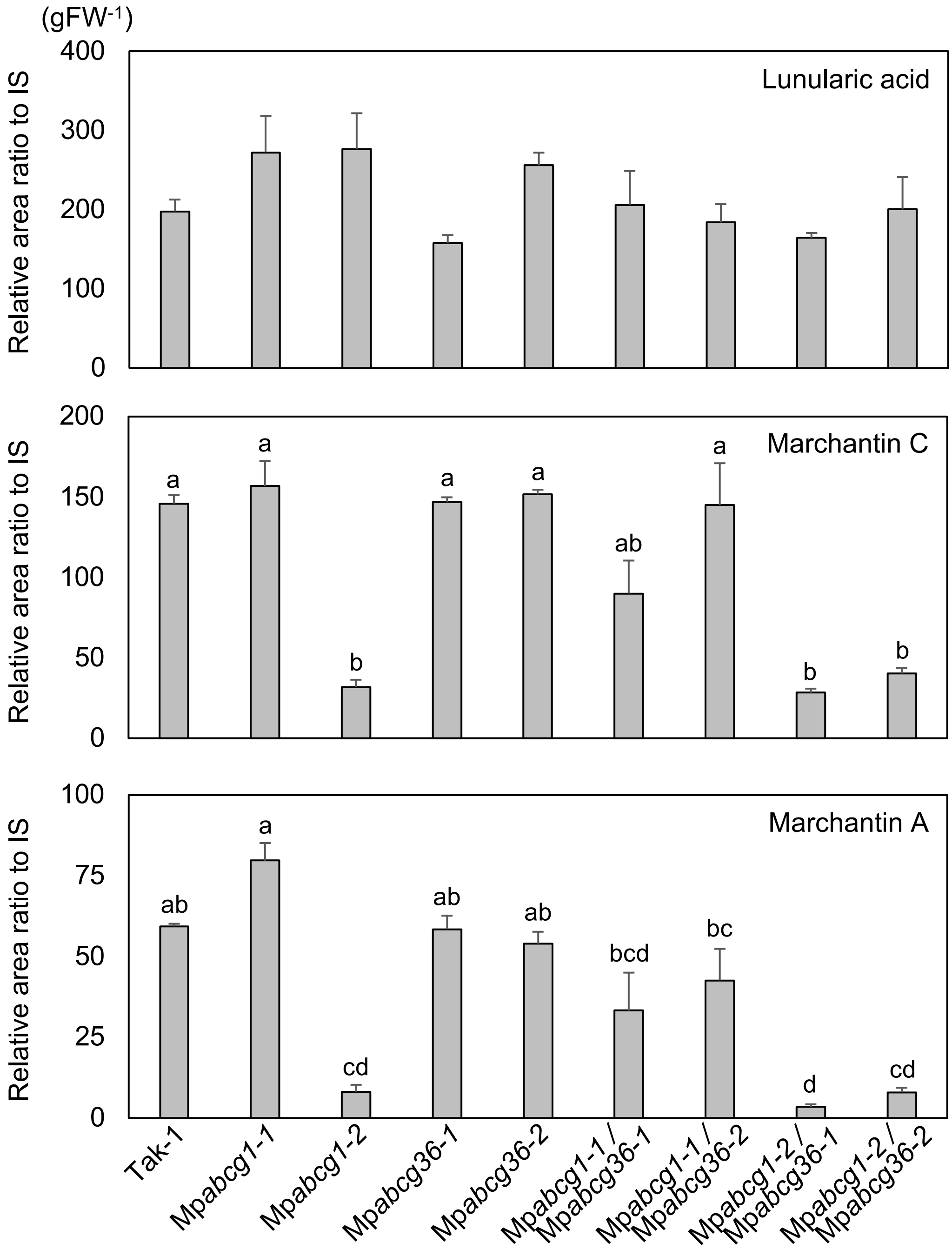

Supplementary Figure S8. Levels of three bibenzyls in all mutants generated in this study.

Levels of three bibenzyls identified in the extracts of the six-week-old thalli of all mutants generated in this study are shown. The relative peak area of each compound normalized to IS (nordihydroguaiaretic acid [NDGA]) is shown. Data are represented as the mean ± SE of three biological replicates. Different lowercase letters indicate the significant differences (one-way ANOVA, followed by Tukey's test; $P < 0.05$).
